# MYB61 is regulated by GRF4 and promotes nitrogen utilization and biomass production in rice

**DOI:** 10.1038/s41467-020-19019-x

**Published:** 2020-10-15

**Authors:** Yihong Gao, Zuopeng Xu, Lanjun Zhang, Shance Li, Shaogan Wang, Hanlei Yang, Xiangling Liu, Dali Zeng, Qiaoquan Liu, Qian Qian, Baocai Zhang, Yihua Zhou

**Affiliations:** 1 State Key Laboratory of Plant Genomics, Institute of Genetics and Developmental Biology, The Innovative Academy of Seed Design, Chinese Academy of Sciences, 100101 Beijing, China; 2 University of Chinese Academy of Sciences, 100049 Beijing, China; 3 Jiangsu Key Laboratory of Crop Genetics and Physiology/Key Laboratory of the Ministry of Education for Plant Functional Genomics, College of Agriculture, Yangzhou University, 225009 Yangzhou, China; 4 State Key Laboratory of Rice Biology, China National Rice Research Institute, Chinese Academy of Agricultural Sciences, 310006 Hangzhou, China

**Keywords:** Agricultural genetics, Natural variation in plants, Cell wall, Plant signalling

## Abstract

Nitrogen (N) is a macronutrient that boosts carbon (C) metabolism and plant growth leading to biomass accumulation. The molecular connection between nitrogen utilization efficiency (NUE) and biomass production remains unclear. Here, via quantitative trait loci analysis and map-based cloning, we reveal that natural variation at the *MYB61* locus leads to differences in N use and cellulose biogenesis between *indica* and *japonica* subspecies of rice. *MYB61*, a transcriptional factor that regulates cellulose synthesis, is directly regulated by a known NUE regulator GROWTH-REGULATING FACTOR4 (GRF4), which coordinates cellulosic biomass production and N utilization. The variation at *MYB61* has been selected during *indica* and *japonica* domestication. The *indica* allele of *MYB61* displays robust transcription resulting in higher NUE and increased grain yield at reduced N supply than that of *japonica*. Our study hence unravels how C metabolism is linked to N uptake and may provide an opportunity to reduce N use for sustainable agriculture.

## Abstract (web summary)

The molecular connection between nitrogen utilization efficiency (NUE) and biomass production is unclear. Here, the authors show that differences in NUE and cellulose biogenesis between rice *indica* and *japonica* subspecies can be explained by variation at the *MYB61* locus, which is regulated by the NUE regulator GRF4.

## Introduction

Carbon (C) and nitrogen (N) are two essential macronutrient elements for plant growth. The large demand of N by plants is in accordance with its importance in producing nucleotides, proteins, chlorophyll, and numerous cellular components, which support fixation of carbon dioxide (CO_2_) to generate sugars, organic acids, and storage carbohydrates^1–3^. All those building blocks sustain plant growth and accumulate as biomass. In the green revolution in the 1960s, supply of inorganic N fertilizer and breed of semi-dwarfism crops that show low responsive to N availability have greatly increased global food production and eliminated world hunger^4,5^. These benefits also cause excess N pollution, which threatens environmental safety and sustainable agriculture^6^. Considering that there is approximately 9 billion people being fed by 2050^7^, improving N-use efficiency (NUE) is still in urgency^3^.

NUE is an inherently complex trait controlled by quantitative trait loci (QTL)^8–11^, in which C and N (C&N) coordination is of the crucial aspect. Plants have evolved an intricate regulatory machinery to coordinate C&N metabolism. One of the controls is at the biochemically metabolic level. Primary N metabolisms, including N uptake, assimilation, and mobilization, significantly affect C fixation and optimize CO_2_ assimilation through controlling photosynthetic and photorespiratory pathways, and vice versa C photosynthetic rates bring impacts on N metabolic fluxes^1,2,12,13^. Arabidopsis nitrate transporters that show varied affinity to nitrate (e.g. NRT1, NRT2) play important roles in N uptake^14,15^, which also seem to act as N sensors required for C assimilation^10,16,17^. Another level for C&N coordination is via signaling and transcriptional regulation. Plants possess distinct C&N sensory systems to monitor the alterations in diverse metabolites^1,18,19^. Numerous signaling components that sense C availability^20,21^, N conditions^9,10,22–24^, and C&N balance^25,26^ have been identified, unraveling a sophisticated regulatory network to orchestrate C&N metabolism. Among these components, GROWTH-REGULATING FACTOR4 (GRF4) is a prominent one due to its wide range of impacts on C&N metabolism in rice^24^. Therefore, the C&N related homeostatic control may be much broader than our expectation.

Nitrogen-mediated plant growth, showing as biomass accumulation, is largely related to plant cell wall biogenesis. There are approximately 56 × 10^9^ metric tons of net CO_2_ being fixed by land plants per year^27^; more than 70% of the CO_2_ assimilated products are converted into polysaccharides, referred to as cell wall polysaccharides, to build the plants themselves^28,29^. Plant cell walls are thus a major sink for the assimilated C partition, which are utilized as constructive and storage materials for plant growth and development, including determining organ morphogenesis and plant architecture, supporting the up-right growth habit, controlling nutrient transduction, stress defenses, etc.^30,31^. All those physiological processes are initiated and sustained by nitrogen availability. However, the known connections are confined to primary C metabolism, such as organic acids, sucrose, and starch synthesis^9,18,24,32^. Although the identifications of Arabidopsis QUASIMODO2/TUMOROUS SHOOT DEVELOPMENT2 and MURUS4 have placed cell wall biogenesis into the interface of C&N responses^33–35^, due to the difficulties in cell-wall characterization, the clear-cut molecule links between cell wall production and nitrogen use remain unclear.

*Indica* and *japonica* are two subspecies of Asian rice (*Oryza sativa* L.) with varied NUE and biomass productivity, in which natural variations contributing to robust NUE and high yield may exist. Rice varieties containing *indica* allele of nitrogen-response regulator *GRF4*^*ngr2*^ and efficient nitrate transporter *NRT1.1B*^*ind*^ manifested the enhanced biomass, which improves NUE and increases grain yield^10,24^. However, the identified gene variations that confer divergent NUE between rice subspecies are still very limited.

In this work, based on the differences in N-mediated leaf area changes and cellulose content in *indica* and *japonica* subspecies, we perform QTL analysis in a set of chromosomal segment substitution lines generated from the two subspecies and identify a QTL coregulating both traits. Gene cloning identifies this locus to a natural variation at *MYB61*, a key transcriptional regulator for cellulose synthesis^36^. We further find that *MYB61* is regulated by GRF4 and GRF4 is pivotal in controlling cellulose biogenesis. The naturally selected *indica* allele of *MYB61* displays robustness in simultaneously governing nitrogen use and biomass production. Our study highlights a connection of N&C interaction at the molecular level and offers a tool for genetic improvement of NUE in rice production.

## Results

### Nitrogen utilization divergence exists in rice subspecies

**Fig. 1:**
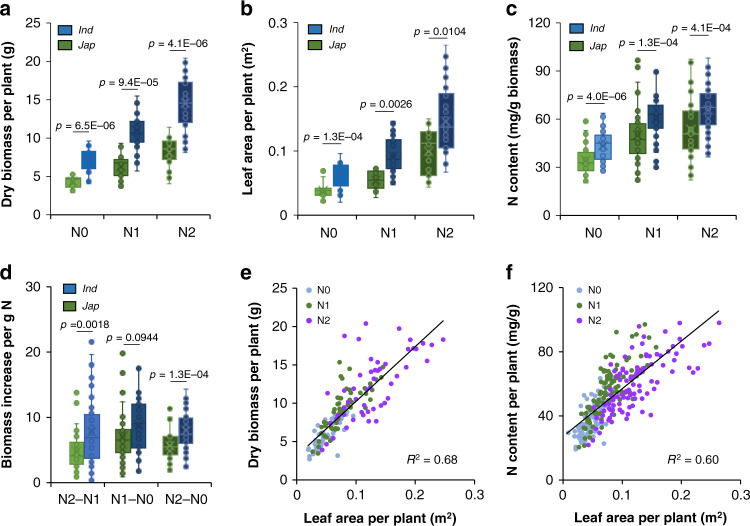
*Indica* and *japonica* subspecies show varied biomass accumulation and nitrogen-use divergence. **a**–**c **Boxplots of the mean amount of dry biomass, leaf area, and total N content of *indica* (*Ind*) and *japonica* (*Jap*) accessions growing under different nitrogen supply at the tillering stage. **d** Boxplot of the mean increment of biomass for each unit of added nitrogen fertilizer of *indica* (*Ind*) and *japonica* (*Jap*) accessions growing under different nitrogen supply at the tillering stage. The mean values in (**a**–**d**) were obtained from nine individual plants of each accession. Box bounds represent the 25th and 75th percentile, center line represents the median, × indicates the mean, and whiskers represent the 25th percentile − 1.5 * the interquartile range and the 75th percentile + 1.5 * the interquartile range. Statistical significance was calculated with two-tailed Welch’s unpaired *t*-test and *p* values are indicated. **e**, **f** Correlation analysis of leaf area with dry biomass (**e**) and with N content (**f**) based on the data shown in (**a**–**c**). N0, N1, and N2 in this figure indicate 0, 96, and 192 (kg/ha) of N, separately. Source data are provided as a Source Data file.

Rice is a major staple cereal crop feeding about three billion people in the world and possesses numerous cultivars adaptation to versatile environments. To discern the robust NUE pyramided in rice varieties, we collected 134 worldwide distributed core rice accessions and planted them in the plots supplied with no N fertilizer (N0), 96 kg/ha (N1), and 192 kg/ha (N2) N supply. The 134 rice accessions including 74 *japonica* and 60 *indica* were resequenced at approximately 15× depth^37^. Phenotyping was conducted in these accessions after cultivation for two months. Based on the obtained data, we found that as the addition of N supply, plant biomass, leaf area, and the N content exhibited increasing trends (Fig. 1a–c). The *indica* varieties consistently generated more amount of biomass and had increased leaf area and N content compared to the *japonica* varieties under the same N supply (Fig. 1a–c). As biomass production is an intuitive performance of NUE, biomass increment of each unit of added N fertilizer that was higher in the *indica* accessions than in the *japonica* accessions (Fig. 1d) further suggested the existence of high NUE in *indica* subspecies. Therefore, the *indica* subspecies may possess natural variations that confer them with robust NUE.

### Identifying QTL for nitrogen-use divergence between *indica* and *japonica*

To identify the variations that contribute to high NUE in *indica*, we performed correlation analysis based the obtained data of rice accessions (Fig. 1a–c). Correlation of leaf area with biomass weight, a known NUE-related trait, and with the N content (Fig. 1e, f and Supplementary Fig. 1) suggested leaf area as one of the N-dependent plant performances. Considering that leaves are a photosynthetic organ and an important place for C&N metabolism, we chose leaf area as an indicator for nitrogen-use divergence in the following QTL analysis.

Nipponbare (NP) and 9311 are two representative cultivars for *japonica* and *indica* subspecies, respectively. In agreement with 9311 that has more biomass than NP (Supplementary Fig. 2a), flag leaf area of the mature 9311 plants was significantly more than that of NP (Supplementary Fig. 2b). Moreover, by treating NP and 9311 seedlings in varied N conditions, 9311 plants had enhanced leaf area and the increases in NP and 9311 was positively correlated with N availability (Supplementary Fig. 2c).

**Fig. 2:**
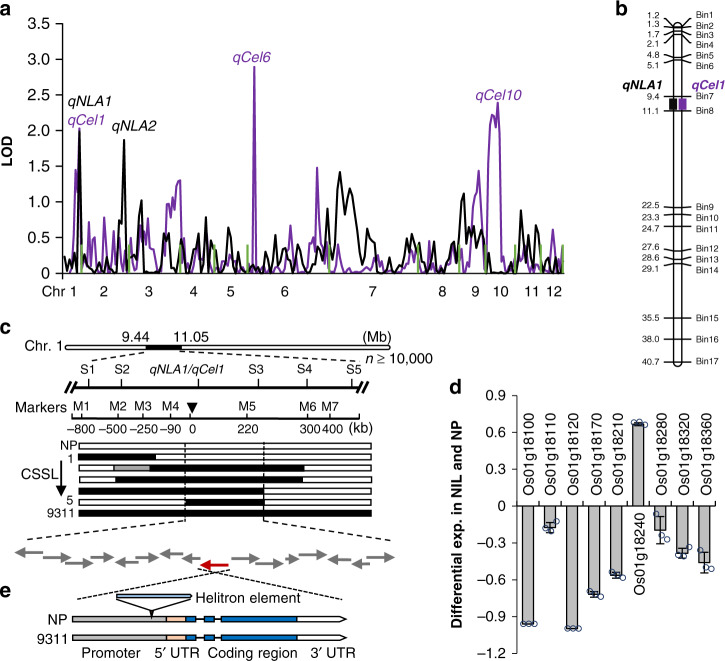
Cloning of *qNLA1/qCel1*. **a** QTL analyses of nitrogen-mediated leaf area changes (*qNLA*, black line) and cellulose content (*qCel*, purple line) in the 9311 introgression lines with NP background. The green lines were used to separate individual chromosomes. **b** *qNLA1* is colocalized with *qCel1*. Different symbols indicate the positions where the corresponding QTL are located. The numbers on the left side of chromosome indicate physical locations (Mb). **c** Mapping of *qNLA1* and *qCel1* in the backcross population. The numbers on chromosome 1 indicate the physical locations. S1–S5 and M1–M7 indicate molecular markers used for mapping. The black and white rectangles represent 9311 and NP genomic region, respectively. Arrows represent candidate genes within the 231.8 kb pin-pointed region according to the performance of the CSSL recombinants (1 to 5) shown in Supplementary Fig. 2f, g. **d** The expression differential ratio of the candidate genes in the NIL to NP plants. Rice *HNR* was used for normalization of the expression of the examined genes. Error bars represent the mean ± SD of three biological replicates. **e** The variation at *Os01g18240* gene. A helitron element is present in the promoter of the NP allele. Source data underlying Fig. 2d are provided as a Source Data file.

We then employed the chromosome segment substitution lines (CSSL) generated by using NP as recipient parent and 9311 as donor parent to conduct QTL analysis. Our previous work has revealed that the 125 core lines cover ~95.6% of the 9311 genome^38,39^. Based on the examinations of leaf area changes in the plants growing under 0.2 mM NH_4_NO_3_ (LN) and 5 mM NH_4_NO_3_ (HN) and the SNP data from the resequenced CSSL population^39^, two logarithm of odds (LOD) score peaks were detected for the leaf area ratio at LN to HN (Fig. 2a), referred as the QTL for nitrogen-mediated leaf area changes (*qNLA1* and *qNLA2*). As the mapping region of *qNLA2* includes the variations at *GRF4* (Supplementary Fig. 2e), a characterized key regulator of NUE^24^, *qNLA1* may contain an unidentified gene and was then subject to gene isolation. By backcrossing (BC) the line possessing *qNLA1* locus with NP, more than 10,000 BC_n_F_2_ and BC_n_F_3_ individuals were generated and subjected to gene mapping using the molecular markers (Fig. 2c and Supplementary Table 1). According to the performance of the resulting CSSL recombinants (Supplementary Fig. 2f), *qNLA1* was narrowed within a 231.8-kb genomic region that contains 16 predicted open reading frames (Fig. 2c). However, none of them were annotated for leaf growth and development.

### *qNLA1* is colocalized with the cellulose locus *qCel1*

Leaf area is highly correlated to biomass accumulation (Fig. 1e); the content of cellulose, a major component of plant biomass, was also higher in parent 9311 than in NP (Supplementary Fig. 2d). We hence examined cellulose content in the internodes, the organ harboring a large proportion of biomass, and characterized QTL for cellulose level (*qCel*) in the same CSSL population. In consequence, three loci *qCel1*, *qCel6,* and *qCel10* were identified (Fig. 2a) and *qCel1* is colocalized with *qNLA1* (Fig. 2a, b). Positional cloning of *qCel1* also narrowed down the mapping region within the 231.8-kb region (Fig. 2c and Supplementary Fig. 2g).

**Fig. 3:**
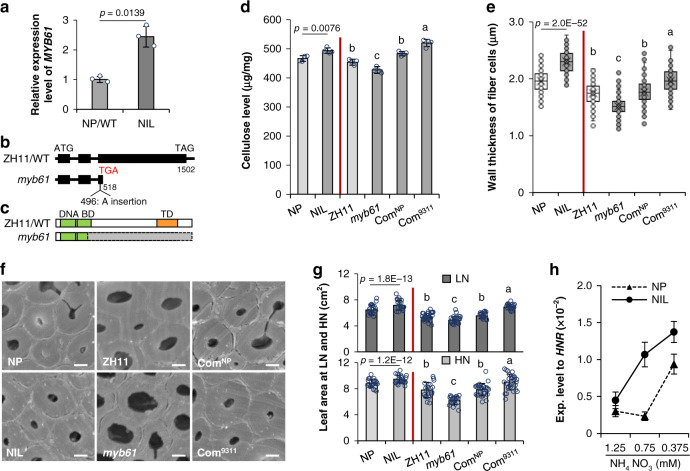
Validation of the *MYB61* function. **a** qPCR analysis of *MYB61* in NP/WT and the NIL containing the 9311-*MYB61* fragment, showing the relative expression to rice *HNR*. Transcription level in NP was set as 1. Error bars represent the mean ± SD of three biological replicates. **b** Schema of *MYB61* gene structure and the mutation site in *myb61*. Boxes and lines in the diagram indicate exons and introns, respectively. **c** Schema of MYB61 protein. Green boxes indicate DNA binding domain (BD) and orange box indicates the transactivation domain (TD). Gray box indicates the truncated part in the mutant. **d** The cellulose content, µg in per mg of cell wall residues prepared from the internodes of the indicated plants. Error bars indicate the mean ± SD of four biological replicates. **e** Boxplot of the wall thickness of sclerenchyma fiber cells in the indicated plants. Box bounds represent the 25th and 75th percentile, center line represents the median, × indicates the mean, and whiskers represent the 25th percentile − 1.5 * the interquartile range and the 75th percentile + 1.5 * the interquartile range. *n* = 200 cells from three individual internodes of the indicated plants. **f** SEM graphs of sclerenchyma fiber cells from the internodes of the indicated plants. Bars = 2 μm. *n* = 3 biologically independent plants. **g** The leaf area of the indicated seedlings growing in the media with LN (0.2 mM NH_4_NO_3_) and HN (5 mM NH_4_NO_3_) supply. Error bars indicate the mean ± SD of at least 19 seedlings. **h** The expression level of *MYB61* in the leaf sheaths of NP and the NIL seedlings treated in varied concentrations of NH_4_NO_3_ for 2 h. Rice *HNR* was used for normalization. Error bars represent the mean ± SD of four biological replicates. Statistical significance was calculated with two-tailed Welch’s unpaired *t*-test and *p* values are indicated in (**a**, **d**, **e**, **g**). Letters a–c in (**d**, **e**, **g**) indicate the different means according to Duncan’s multiple range test (*p* < 0.05). Com^NP^ and Com^9311^ represent the plants expressing the complementary constructs shown in Supplementary Fig. 2e. The red vertical lines in (**d**, **e**, **g**) were used to separate the plants with different genetic background. NP, Nipponbare; NIL, near-isogenic line; ZH11, Zhonghua11. Source data underlying Fig. 3a, d, e, g, h are provided as a Source Data file.

To clone the corresponding gene, we investigated the transcripts of the genes within the mapping region in the internodes. Among the 16 ORFs, nine of them are broadly expressed based on the RNA sequencing data of the NP developing internodes (Supplementary Fig. 2h). Quantitative PCR (qPCR) was further performed to compare the expression level of these genes in NP and a near-isogenic line (NIL) that contains the 231.8 kb of 9311 fragment. We found that *Os01g18240* is the only gene being upregulated in the NIL (Figs. 2d and 3a), in consistence with the more cellulose level and increased flag leaf area and internode diameter in the NIL (Fig. 3d and Supplementary Fig. 3a–d). *Os01g18240* encodes a master transcriptional factor *MYB61* controlling cellulose biosynthesis^36^. Sequencing analysis revealed that a helitron element, a transposon previously annotated as hel_osa_val_0065^40^, is present in the promoter region of the NP allele, whereas no variation was found in the coding region (Fig. 2e). Therefore, *Os01g18240/MYB61* may be the candidate of *qNLA1/qCel1*.

### The variation at *MYB61* mediates divergence in nitrogen use and biomass

To verify the above hypothesis, we generated a *myb61* mutant by CRISPR/Cas9 approach in a *japonica* wild-type variety Zhonghua11 (ZH11). One base-pair insertion was found located at the 496 site of *MYB61*, which results in a stop codon and disrupts its transcriptional activation domain (Fig. 3b, c). The *myb61* mutant plants exhibited reduced cellulose level (Fig. 3d), in agreement with our previous finding that overexpression of *MYB61* improved cellulose synthesis^36^. The *myb61* mutant also displayed reductions in flag leaf area and internode diameter compared to the wild-type variety ZH11 (Supplementary Fig. 3b–d). We further conducted the genetic complementary analysis by expressing *MYB61*^NP^ promoter or *MYB61*^9311^ promoter driving *MYB61* (Com^NP^ or Com^9311^) in the *myb61* mutant plants (Supplementary Fig. 3f). The complementary constructs rescued the decreased cellulose content to the ZH11 (wild type) level (Fig. 3d), which was corroborated by the evidence that the wall thickness of the mutant internode fiber cells became comparable to ZH11 as revealed by scanning electronic microscopy (SEM) analysis (Fig. 3e, f). Moreover, Com^9311^ rescued the wall thickness and cellulose content to a high level compared to Com^NP^, in accordance with the finding that the NIL harboring the 9311 allele of *MYB61* displayed higher cellulose level and more thickened wall in fiber cells than the recurrent parent Nipponbare (NP) (Fig. 3d–f). In addition, qPCR examination in several organs detected abundant *MYB61* transcripts in the developing internodes and panicles (Supplementary Fig. 4a), suggesting that *MYB61* is one of the dominant genes regulating cellulose biosynthesis in rice.

To determine whether MYB61 contributes to the nitrogen-use divergence, the transgenic seedlings were cultivated in a media containing 0.2 mM NH_4_NO_3_ (LN) or 5 mM NH_4_NO_3_ (HN). We found that leaf area at LN and HN in the NIL was increased compared to the recurrent parent NP (Fig. 3g); both complementary constructs recovered the reduced leaf area of *myb61* to ZH11 (wild type) level (Fig. 3g). Taken together with the reduced NUE in *myb61* plants (Supplementary Fig. 3e) and the consistent alteration tendency in leaf area ratio and biomass abundance at LN and HN (Supplementary Fig. 3g, h), *MYB61* appears to affect nitrogen utilization. By performing qPCR analysis in NP and the NIL plants, we further found that *MYB61* expression is induced by low N supply (Fig. 3h), similar to the expression pattern of N homeostasis genes, e.g. *GRF4*, *AMT1.1*, and *NRT2.1*^41,42^ under the limited N condition (Supplementary Fig. 4b). Moreover, the 9311 allele of *MYB61* seemed responsive to low N availability more robustly than the NP allele (Fig. 3h).

Therefore, the variation at *MYB61* contributes to the divergence in nitrogen use and cellulosic biomass production between 9311 and NP varieties.

### Integrating GRF4 into the *MYB61* regulatory pathway

**Fig. 4:**
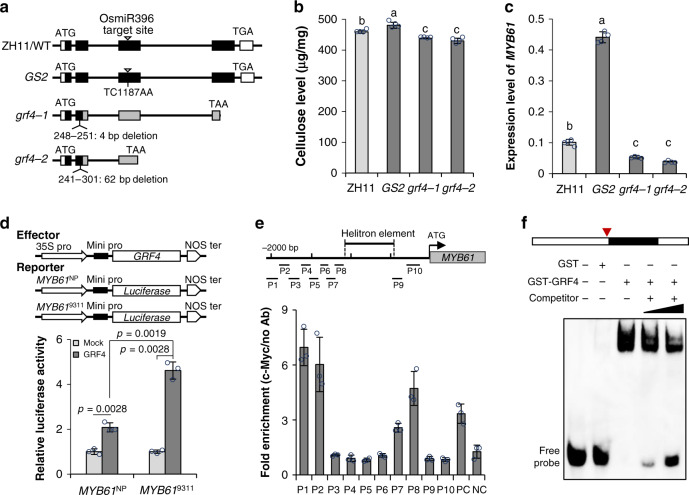
GRF4 is involved in cellulose biogenesis via regulating *MYB61* transcription. **a** Schema of *GRF4* gene structure and the mutation sites of *GS2* and *grf4* mutants. Boxes and lines in the diagram indicate exons and introns, respectively. Gray boxes indicate mutated parts in the mutants. **b** The cellulose content, µg in per mg of cell wall residues prepared from the internodes of the indicated plants. Error bars indicate the mean ± SD of four biological replicates. Letters a–c indicate the different means according to Duncan’s multiple range test (*p* < 0.05). **c** The expression level of *MYB61* in the leaf sheaths of indicated plants. Rice *HNR* was used for normalization. Error bars represent the mean ± SD of four biological replicates. Letters a–c indicate the different means according to Duncan’s multiple range test (*p* < 0.05). **d** Transcriptional activation assays by cotransfecting Arabidopsis protoplasts with the constructs shown in the upper panel. The luciferase activity was normalized with that of the cells coexpressing the empty effector (Mock). Error bars represent the mean ± SD of three biological replicates. Statistical significance was calculated with two-tailed Welch’s unpaired *t*-test and *p* values are indicated. **e** ChIP-PCR enrichment of the GRF4-cMyc targeting DNA fragments in the *MYB61* promoter region. The upper panel indicates the locations of the DNA fragments subjected to ChIP-PCR analysis. Error bars represent the mean ± SD of three biological replicates. Rice glutamine synthase *GS1.2* was used as the positive control (PC); an exon of *MYB61* was used as the negative control (NC). **f** EMSA assay, showing the GRF4 recombinant proteins bound to the motif neighboring to the helitron element insertion site at the *MYB61* promoter region. The open and black boxes indicate the *MYB61* promoter and the helitron element, respectively. The red triangle indicates the site for generating the probe. *n* = 3 independent experiments. Source data underlying Fig. 4b–f are provided as a Source Data file.

Next, we investigated how *MYB61* links to nitrogen-use divergence at the molecular level. As GRF4 is an identified regulator controlling NUE^24^ and located in the region of *qNLA2* (Fig. 2a), we hypothesized that *MYB61* and *GRF4* may share common regulatory pathways. To verify this hypothesis, the *grf4* mutants were generated by CRISPR/Cas9 method. Two loss-of-function mutants (*grf4-1* and *grf4-2*) were obtained, in which a 4 bp or a 62 bp deletion that results in a frameshift and prematurely translational products was found at the site of 248–251 bp and 241–301 bp, respectively (Fig. 4a). In addition, a previously reported mutant *GS2*, in which the replacement of TC with AA disrupts the OsmiR396 binding site and causes accumulation of *GRF4* transcripts^43–45^, was used as a gain-of-function mutant. Phenotyping these mutants showed that the gain-of-function mutant *GS2* had larger flag leaves and thicker internodes than the wild-type variety ZH11, whereas reduced flag leaf and internode diameter were observed in the loss-of-function mutants *grf4-1* and *grf4-2* (Supplementary Fig. 5a–c). More importantly, scanning electron microscopy and compositional analysis revealed thicker cell wall and more cellulose content in *GS2* and thinner wall thickness and less cellulose level in *grf4-1* and *grf4-2* compared to ZH11 (Fig. 4b and Supplementary Fig. 5d, e). All those demonstrated that GRF4 is indeed involved in the control of biomass and cellulose production. By performing qPCR analysis in these mutants, we found that *MYB61* is highly expressed in the gain-of-function mutant *GS2*, but is significantly repressed in the two loss-of-function mutant *grf4-1* and *grf4-2* (Fig. 4c). Hence, GRF4 controls cellulose synthesis probably via regulating *MYB61* transcription.

To verify this hypothesis, we cotransfected the effector *Pro35S*:GRF4 with the reporter containing the NP- or 9311-*MYB61* promoter driving *luciferase*, *Pro MYB61*^NP^:LUC or *Pro MYB61*^9311^:LUC, in Arabidopsis protoplasts (Fig. 4d). By normalizing the activities of the protoplasts cotransfecting the empty reporter construct (mock), the luciferase activities promoted by coexpressing GRF4 and *Pro MYB61*^9311^:LUC were one fold higher than those activated by expressing GRF4 and *Pro MYB61*^NP^:LUC (Fig. 4d). Hence, GRF4 is an upstream regulator of *MYB61* and the 9311 allele of *MYB61* is more active. Since the *MYB61* promoter of NP and 9311 possess GRF4 binding motifs (Supplementary Fig. 6a), we next explored whether GRF4 can bind to these motifs in vivo. By expressing GRF4-cMyc in the *grf4* mutant, chromatin-immunoprecipitation analysis (ChIP) was conducted. ChIP-PCR analysis revealed three associations between GRF4 and the *MYB61* promoter segments at P1, P2, and P8 (Fig. 4e). Electrophoretic mobility shift assays (EMSA) further demonstrated the binding of glutathione *S*-transferase (GST)-tagged GRF4 to the *MYB61* promoter fragment at P8 containing motif 2 (Fig. 4f and Supplementary Fig. 6a), but no bound signal was detected by using P1 and P2 fragments and the fragment harboring motif 1 as probes (Supplementary Fig. 6). It should be noted that the confirmed binding site at P8 is closely adjacent to the helitron insertion place (Fig. 4f and Supplementary Fig. 6a). Therefore, GRF4 directly regulates *MYB61* transcription.

### Evidence of selection at *MYB61* locus

**Fig. 5:**
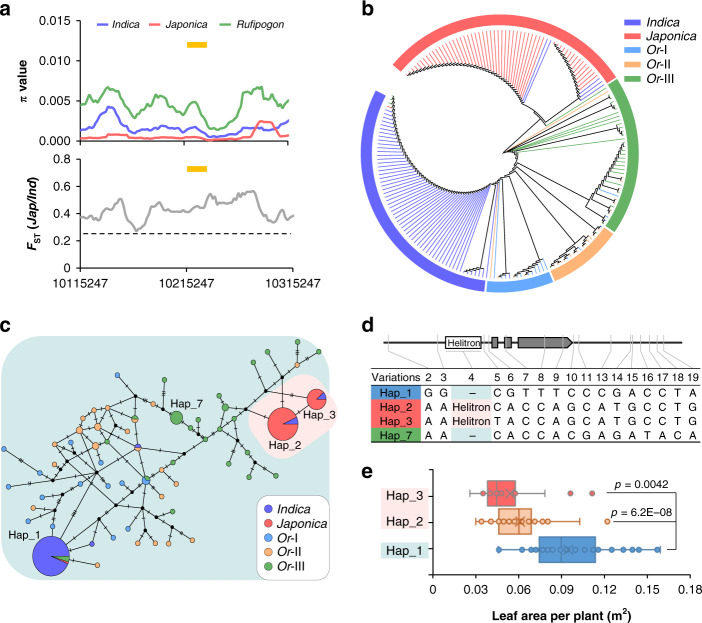
Domestication of *MYB61* in rice. **a** *π* and *F*_ST_ values between *indica* and *japonica* in the 200 kb genomic region containing the *MYB61* locus (the yellow line). The dashed line indicates the genome-wide threshold. **b** Phylogenetic tree of the *MYB61* genomic sequences in the examined accessions. *Or*, *O. rufipogon*. **c** Phylogenetic network of *MYB61* with pink and light green backgrounds denoting haplotypes containing the helitron insertion or not. The node size is proportional to the sample size. **d** Sequence alignment of *MYB61* variations in the examined accessions. Blue background indicates haplotype in *indica*, red background indicates the haplotypes in *japonica* and green background indicates the haplotypes in common wide rice. The variations were placed on the schema of the *MYB61* gene. **e** Boxplot of the leaf area of the rice accessions with haplotype_1 to _3. The mean value was obtained from nine individual plants of each accession. Box bounds represent the 25th and 75th percentile, center line represents the median, × indicates the mean, and whiskers represent the 25th percentile − 1.5 * the interquartile range and the 75th percentile + 1.5 * the interquartile range. Statistical significance was calculated with two-tailed Welch’s unpaired *t*-test and *p* values are indicated. The indication of pink and light green background in (**d**, **e**) is the same as in (**c**). Source data are provided as a Source Data file.

To investigate whether *MYB61* has undergone selection during rice domestication, we analyzed sequence variations at *MYB61* in 42 common wild rice accessions and the 134 cultivated accessions because these accessions have successfully employed in gene domestication analysis^37^. Compared to wild rice accessions, nucleotide diversity (π) of *MYB61* in *indica* and *japonica* cultivars was lower (Fig. 5a). Meanwhile, the *F*_ST_ value that indicates genetic divergence in *MYB61* was obviously above the genome-wide threshold 0.27 (Fig. 5a), suggesting a great difference between *indica* and *japonica* populations. These results indicated the occurrence of selection in this gene, which was further corroborated by the negative Tajima’s D value at the region of *MYB61* compared to its adjacent sequences (Supplementary Fig. 7a). To evaluate *MYB61* variation, 56 additional *O. rufipogon* (*Or*) accessions classified into *Or*-I, *Or*-II, and *Or*-III^46^ were included with the 176 rice accessions. The genomic fragments containing the 3 kb promoter, gene coding region, and 3 kb of terminator regions of the 232 accessions were subjected to determine the variation at *MYB61*. A phylogenetic tree was built based on the SNPs within the *MYB61* genomic fragments; the *indica* and *japonica* cultivars were grouped into different clades and adjacent to the clades containing their progenitor *Or*-I and *Or*-III accessions, respectively (Fig. 5b). A few miscellaneous accessions may arise from gene introgression during breeding. All those suggested that *MYB61* diverged in *indica* and *japonica* subspecies.

Moreover, eleven haplotypes presenting in at least two rice accessions were identified, in which Hap_1–3 and Hap_7 are the major haplotypes (Fig. 5c). The common wild rice ecotypes possess diversified haplotypes including Hap_7, whilst most *indica* accessions harbor Hap_1. In addition to that Hap_2 and Hap_3 are mainly present in the *japonica* accessions, the helitron transposon^40,47^ was found present at the promoter of *MYB61* in all examined *japonica* varieties, but absent there in the examined *indica* and common wild rice accessions (Fig. 5c, d). PCR analysis further verified that this insertion also does not exist in the examined wild rice accessions with CC and EE genome (Supplementary Fig. 7b). Furthermore, we found that the SNP (A to T) in Hap_1, which results in a missense variation (Ser337Thr) in the coding region, is only present in the *indica* varieties. However, the absence of this variation in 9311 ignored it as a divergence in this study. More importantly, the *indica* cultivars that possess Hap_1 allele (in absence of the helitron insertion) exhibited increased leaf area, enhanced biomass production, and more N content compared to the *japonica* cultivars that harbor Hap_2 and Hap_3 alleles (in presence of the helitron insertion) (Fig. 5e and Supplementary Fig. 7c). Therefore, the difference in biomass production in *indica* and *japonica* is conferred by the variations at *MYB61* to some extent.

Taken together, the variation at *MYB61* likely occurred while Asian cultivated rice was domesticated into *indica* and *japonica* from the common wild rice.

### The *indica* allele of *MYB61* promotes biomass production and grain yield

**Fig. 6:**
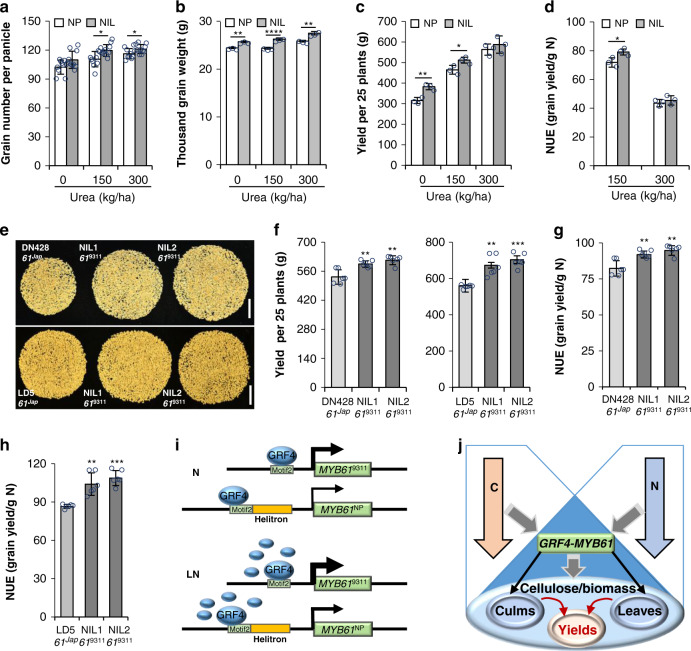
The 9311 allele of *MYB61* shows enhanced NUE and grain yield. **a**, **b** Measurement of the agronomic traits of NP and the NIL growing in the fields under different urea conditions. Error bars indicate the mean ± SD of 10 plants in (**a**) and three biological replicates in (**b**). **c** Grain yield of 25 plants of NP and the NIL growing under the indicated urea supply. Error bars indicate the mean ± SD of three biological replicates. **d** NUE of NP and the NIL growing under the indicated urea condition. Error bars indicate the mean ± SD of three biological replicates. **e** Grains per plant of the recipient varieties and the NILs growing under 150 kg/ha urea condition. Bar = 5 cm. *n* = 3 biologically independent plants. **f** Grain yield of 25 plants of the recipient varieties and the NILs growing under 150 kg/ha urea condition. Error bars indicate the mean ± SD of six biological replicates. **g**, **h** NUE of the recipient varieties and the NILs growing under 150 kg/ha urea condition. Error bars indicate the mean ± SD of six biological replicates. **i** The working model of GRF4-regulated *MYB61*. GRF4 binds to *MYB61* promoter region; however, the helitron element insertion compromises GRF4 binding and transactivation activity. Low nitrogen triggers GRF4 accumulation, which enhances *MYB61* transcription. The expression level of *MYB61* is proportional to the thickness of arrows. **j** The metabolic fluxes mediated by GRF4 and MYB61. The GRF4-MYB61 model coordinates C&N metabolism, which promotes cellulosic biomass production and grain yield. Statistical significance in this figure was calculated with two-tailed Welch’s unpaired *t*-test and *p* values are indicated (*p < 0.05, **p < 0.01, ***p < 0.001, ****p < 0.0001). Source data underlying Fig. 6a–d and 6f–h are provided as a Source Data file.

We next undertook the field trials to compare the agronomic traits of the NIL and its recurrent parent NP growing in the fields supplying with 0 kg/ha, 150 kg/ha, and 300 kg/ha of urea. The NIL harboring the 9311 allele of *MYB61* displayed significant growth robustness over NP, showing as the increases in biomass production (Supplementary Fig. 8a–c) and resulting in more grain number per panicle and the augmented grain weight (Fig. 6a, b). Consequently, grain yield per 25 plants of the NIL was increased about ~21.4% and ~10.1% over NP under 0 and 150 kg/ha urea condition, respectively, but was almost invariant by supplying 300 kg/ha urea (Fig. 6c). NUE in the NIL was improved at low N supply but remained unchanged at high N condition (Fig. 6d). Hence, *MYB61*^9311^ can enhance NUE and grain productivity, especially at low N condition.

Moreover, the 9311 allele of *MYB61* was introduced into Dongnong428 (DN428) and Longdao5 (LD5), two major *japonica* cultivars widely cultivated in the northeast of China. Through backcross plus maker assistant selection, two NILs selected from BC_4_F_5_ lines were subjected to field trials. Under 150 kg/ha of urea supply, these NILs exhibited increases in plant height and biomass compared to the corresponding recipient parents (Supplementary Fig. 8d, e). Grain number was consistently augmented in these NIL lines (Fig. 6e), leading to 11.7–15.1% (in DN428) and 20.1–25.7% (in LD5) yield increases (Fig. 6f) and significantly enhanced NUE (Fig. 6g, h) compared to the recipient parents.

To further investigate the potential breeding utilization of GRF4 and MYB61, a NIL that pyramids 9311 alleles of *GRF4* and *MYB61* was generated by genetically crossing the NILs containing either of the 9311 alleles. The agronomic traits were measured in the mature plants growing in the field with 150 kg/ha urea supply. Consequently, pyramiding 9311 *GRF4* and *MYB61* significantly enhanced biomass, gain number and grain yield compared to the plants possessing none or either 9311 allele (Supplementary Fig. 9). Moreover, NUE was obviously improved in the NILs containing either or both alleles (Supplementary Fig. 9i), whereas harvest index was not affected (Supplementary Fig. 9j).

Therefore, the *indica* allele of *MYB61*, as well as the combination of *indica GRF4* and *MYB61*, represents elite variations conferring improved NUE, which promoted robust biomass production especially under limited N condition.

## Discussion

N and C are two basic constructive elements. Plants uptake N from soils and fix CO_2_ from atmosphere; both elements are converted into diverse metabolites through C&N metabolisms that are balanced via an elaborate coordination machinery. Here, we identified a QTL that coordinates C&N metabolisms and controls NUE and biomass production. A series lines of evidence demonstrated that a natural variation at *MYB61* represents one of the key determinants governing cellulosic biomass biogenesis and nitrogen-use divergency between rice subspecies. *MYB61*, which expression is regulated by GRF4, is a crucial nexus to coordinate C&N metabolisms in rice.

The *indica* varieties often display higher NUE than the *japonica* varieties^48^. Increasing studies revealed that robust NUE commonly exhibits superiority in biomass biogenesis, showing as increased plant height, more tillers, and high grain yield^9,10,49–51^. Therefore, the high NUE is associated with enhanced biomass accumulation; but how N promotes biomass production is so far unclear. Cellulose usually constitutes ~40–90% of plant cell wall biomass^52,53^. In this study, we performed QTL analysis of nitrogen-use divergency in a CSSL population that was generated by introgressing an *indica* accession 9311 genome into a *japonica* accession NP. By examining N-mediated leaf area changes and cellulose content in this population, we found several QTL, in which *qNLA1* and *qCel1* are colocalized. Gene cloning revealed that the *qNLA1*/*qCel1* resulting differences are caused by the variation at *MYB61*, a key regulatory factor for cellulose biosynthesis^36^. This conclusion was further corroborated by the leaf area and cellulose content performances of several genetic materials, including *indica-MYB61* NIL, the *myb61* mutant, and the complementary transgenic plants. Therefore, the *MYB61* variation confers 9311 and NP with divergence in nitrogen use and biomass production. Nucleotide diversity and haplotype analyses in around two hundreds of rice accessions identified additional variations in this gene, and suggested that *MYB61* is selected during rice domestication. Among these haplotypes, the absence of a helitron transposon in the *MYB61* promoter region was found in the examined *indica* varieties, common wild rice with AA genome, and wild rice accessions with CC and EE genomes; this insertion was present in the examined *japonica* varieties, suggesting it as a representative variation in the overall *indica* and *japonica* varieties. By searching in the five available resequenced high-quality genomes (*japonica*: NP and Kitaake; *indica*: IR8, R498 and 9311), none of other helitron was found to share the same sequence with this transposon except with ~99% sequence similarity (Supplementary Table 2), implying that this insertion has been fixed in *japonica* during rice domestication. The high sequence similarity in these transposons further indicated that they may share the same origin. Hence, *MYB61* likely proceeded this selection while *indica*-*japonica* divergence, in agreement with the reports that helitron transposon participates in *indica*-*japonica* evolution^47^. Of course, further studies are needed to clearly address the origin and characteristics of this helitron, as well as the selection route of *MYB61*. Thus, *MYB61* is a critical gene connecting C&N metabolism; our study offers the molecular link between nitrogen-use divergency and biomass biogenesis.

The further supports for *MYB61* connecting C&N metabolism came from the findings that *MYB61* is responsive to low N treatments and regulated by rice GRF4, an integrative regulator of multiple N metabolism genes, as well as a coordinator of C metabolism^24^. Characterizations of QTL for high NUE in rice have identified natural variations of *GRF4*, which enhanced C&N assimilation and biomass production, conferring the rice varieties with increased yield^24,44^. In this study, we also identified *qNLA2* that harbors 9311 allele of *GRF4*. We further found that the *GRF4* gain- and loss-of-function mutants showed corresponding alterations in biomass production and cellulose content. The transcription of *MYB61* was upregulated in the *GRF4* gain-of-function mutant but was suppressed in the loss-of-function mutants, which placed GRF4 upstream of *MYB61*. The transactivation activity assays and analyses of ChIP-PCR and EMSA verified this conclusion. Moreover, the GRF4 binding motif that is adjacent to the helitron transposon insertion was identified. The helitron transposon insertion may interrupt GRF4 binding and compromise *MYB61* transcription, otherwise would benefit the transcriptional activity (Fig. 6i). Although GRF4 has been revealed as a C&N integrator with broad impacts on plant growth, its role in regulating *MYB61* transcription and controlling cellulosic biomass production has not been characterized.

Under the requirement of reducing N fertilizer application, improving NUE is essential for modern sustainable agricultural production. The inducible performance of GRF4 at limited N^24^ promotes accumulation of this protein at low N availability, which regulates the transcription of the *indica* and *japonica MYB61* alleles at varied activities (Fig. 6i). The NIL containing 9311 allele of *MYB61* thus exhibited more biomass production under the limited N condition, resulting in increased yield and NUE at low N supply compared to NP. Introduction of the 9311 allele into the elite *japonica* cultivars DN428 and LD5 also significantly enhanced biomass production, as well as grain yields and NUE, suggesting a broad value of this *indica* allele in improving NUE in a wide range of *japonica* varieties. Pyramiding 9311 *GRF4* and *MYB61* alleles in the NIL further enhanced biomass, grain yield, and NUE compared to the plants possessing none or either 9311 allele. Therefore, GRF4 and MYB61 may constitute a regulatory cascade, which represents one of the coordinators in C&N metabolism that simultaneously govern NUE and cellulosic biomass production (Fig. 6j), and could be applied in rice molecular breeding. Our study hence uncovers the mechanism for C&N coordination and provides a solution to maintenance of grain yield while reducing agricultural N use in crop production.

## Methods

### Plant materials and growth conditions

The 134 core rice accessions were collected and supplied by State Key Laboratory of Rice Biology, China National Rice Research Institute, Chinese Academy of Agricultural Sciences. A total of 18 plants of each accession were planted in a completely randomized blocks that were designed with three replicates under different N fertilizer condition at the experimental station of China National Rice Research Institute in Hangzhou (Zhejiang Province). The three nitrogen fertilizer levels were N0 (no N supply), N1 (96 kg/ha N), and N2 (192 kg/ha N). Urea is the only source of nitrogen. The plants were cultivated with a distance of 25 × 15 cm.

The NP-9311 CSSL population was developed by Key Laboratory of the Ministry of Education for Plant Functional Genomics, College of Agriculture, Yangzhou University^39^. The 125 CSSL were planted with a distance of 25 × 15 cm at the experimental fields of Yangzhou University in Yangzhou (Jiangsu Province) in the summers of 2015–2019. The 2nd internodes were harvested from the mature plants and subjected to cellulose content analysis. For examination of the leaf area changes, the seeds of 125 CSSLs were sown and cultivated in the hydroponic media containing 0.2 mM NH_4_NO_3_ or 5 mM NH_4_NO_3_ in a growth chamber (16 h light at 28 °C and 8 h dark at 26 °C with 70% humidity) until the fourth leaf emerging. AgriPheno High Throughput Plant Genotyping-Phenotyping-Breeding Service Platform was employed to measure the leaf area. Easy Leaf Area Software was used to quantify leaf area^54^.

The NILs were generated by backcrossing the CSSL containing the 9311 allele of *MYB61* with three *japonica* varieties, NP, DN428, and LD5, respectively. With the aids of maker assistant selection and phenotyping, the NILs were obtained from BC_4_F_5_–BC_7_F_3_ and confirmed by genome resequencing or maker characterization. The NIL pyramiding *MYB61*^9311^ and *GRF4*^9311^ was generated by genetically crossing the NILs containing either of the 9311 alleles. The NIL plants with NP background were cultivated in paddy fields supplied with 0, 150 kg/ha, and 300 kg/ha urea in the experimental station of the Institute of Genetics and Developmental Biology, Beijing in 2018–2019. The NIL plants with DN428 and LD5 background were cultivated in the paddy fields under 150 kg/ha urea condition in Harbin (Heilongjiang Province) in 2018–2019. The NIL plants pyramiding both or either 9311 alleles were cultivated in the fields with 150 kg/ha urea supply at the experimental station in Lingshui (Hainan Province) and the experimental station of China National Rice Research Institute in Hangzhou (Zhejiang Province). Therefore, the field experiments were conducted for two years and in each year at least three replicates were included.

For generation of *myb61* and *grf4* mutants by CRISPR/Cas9, the coding sequences were chosen as the target sequences and cloned into the binary plasmid (pYLCRISPR/Cas9 Pubi-MH)^55^. For complementary analysis, the promoter fragments of *MYB61* were amplified from NP and 9311 genomic DNA, and cloned into the binary vector pCAMBIA1300, respectively. The *MYB61* cDNA was amplified and inserted into the resulting vector between the *MYB61* promoter and *NOS* terminator. The transgenic plants were generated by *A. tumefaciens* strain EHA105 infection^56^ and grew in the experimental fields at the Institute of Genetics and Developmental Biology in Beijing and in Lingshui (Hainan Province) in different growth seasons. The primers used for amplification were listed in Supplementary Table 3.

### Field trials

A randomized block design approach was applied in the analyses of agronomic traits. To evaluate nitrogen-use divergency in *indica* and *japonica* accessions, we measured the agronomic traits, including plant height, leaf area per plant, and dry biomass per plant, in the 134 core rice accessions that grew in the fields with different N supply at the tillering stage. Meanwhile, total nitrogen contents were measured by dissipating biomass in a drying oven and detecting using the Kjeldahl method^57^. Consequently, the complete data from 97 *indica* and *japonica* core varieties were used for statistics and correlation analysis. The NILs with three *japonica* background were cultivated in randomized plots supplied with varied amounts of urea. The plant height, flag leaf area, tiller number, grain number per panicle, and 1000-grain weight were investigated in nine representative individual plants. After harvest, seeds derived from 25 individual plants from each plot were pooled and subjected to measure the grain yield. NUE was determined by the ratio of total grain yield to applied N fertilizer. The field trials were conducted for at least two years.

### Cellulose examination

The 2nd internodes were harvested from at least 15 mature plants of 125 CSSLs. Alcohol-insoluble residues were prepared by pooling the ground internodes of each line. After destarched with α-amylase (Megazyme) in MES-Tris buffer (pH 8.1), the remains were treated with Updegraff reagent (acetic acid:nitric acid:water, 8:1:2, v/v) to remove noncellulosic sugars. The insoluble samples were hydrolyzed by 72% (v/v) sulfuric acid. The cellulose content was examined using the anthrone assay^56^. Four biological replicates were included in the examination.

### QTL mapping and gene cloning

Based on the phenotypes and the SNPs obtained by the resequenced 125 CSSL^40^, the QTL analysis was performed using ICIMapping 4.0 software with a likelihood ratio test based on single-point analysis. LOD larger than 1.5 were considered as QTL. To clone the candidate gene, more than 10,000 BC_n_F_2_ and BC_n_F_3_ plants were developed by backcrossing the CSSL containing *qNLA1* with the recurrent parent NP. Thirteen DNA makers were developed for fine mapping the *qNLA1*/*qCel1* (Supplementary Table 1).

### Gene expression analysis

The 9-cm developing internodes were harvested from NP and were cut into nine segments. Total RNA was extracted from each segment and subjected to RNA sequencing according to the manufacturer’s instructions (Berry Genomics). For qPCR analysis, different organs, including roots, leaf sheaths, and leaves of 2-week-old seedlings, the 9-cm developing internodes, and young panicles ranging in 0.5–20 cm long were collected from NP to isolate the total RNA using Plant RNA Reagent (Invitrogen). Approximately 2 µg of the total RNA was treated with DNase I to synthesize the first-strand cDNA using oligo (dT) as a primer. The double-strand cDNA was obtained using the PrimeScript RT Reagent Kit (TAKARA). qPCR reactions labeled by FastStart Universal SYBR Green Master (Roche) were performed on a cycler apparatus (Bio-Rad CFX96). The data were analyzed by the 2-DCT method. Rice *HNR* and *Actin* were used as internal controls. To examine the expression of *MYB61* in *GS2* and *grf4* mutants, the leaf sheaths from 3-week-old rice seedlings were subjected to total RNA isolation. To determine whether *MYB61* is induced by low N, the 2-week-old NP seedlings were treated in the media containing varied concentrations of NH_4_NO_3_ for 2 h. qPCR analyses were conducted as described above. The qPCR primers were listed in Supplementary Table 4. Three biological replicates were included.

### Transactivation activity analysis

The full-length coding sequence of *GRF4* was amplified and cloned into the vector p2GW7 to generate the effector construct. The reporters *Pro MYB61*^*NP*^:LUC or *Pro MYB61*^*9311*^:LUC were prepared by amplifying the *MYB61* promoter from NP and 9311 and inserting them into the pUC19 vector that contains the luciferase gene. The primers for PCR amplification were shown in Supplementary Table 3.

Transactivation analysis was performed in the protoplasts extracted from 4-week-old Arabidopsis rosette leaves^36^. By incubating the transfected protoplasts for overnight, the cells were lysed and subjected to detect the luciferase activity according to the instruction of the Dual-Luciferase Reporter Assay System (Promega, E1960). The *Renilla reniformis* luciferase gene driven by CaMV 35S was used as an internal control. This analysis was repeated for three times.

### ChIP-PCR

The dehulled seeds of *grf4* mutants were sterilized with 30% Kao bleach (Japan) for 1 h. After thorough rinse with sterile water, the seeds were incubated on 1/2 MS medium in an incubator with a 16 h light/8 h dark photoperiod at 26 °C for 10 days. Rice protoplasts were prepared from 2-week-old *grf4* seedlings that were cultivated on the 1/2 MS media^58^. 500 μL protoplasts solution containing about 2 × 10^7^ cells were mixed with 80 μg of the GRF4-cMyc plasmid DNA, and subjected to PEG-mediated transfection. The transfected protoplasts were cultivated under dark at room temperature for 16–20 h. Protoplasts were harvested and cross-linked with 1% (v/v) formaldehyde for 10 min. The reactions were stopped by adding glycine to 0.1 M and further incubated for 5 min. After lysis of the nuclei, the DNA was sheared into fragments with an average size of ~300 bp by sonication. The resulting samples were centrifuged at 12,000 g for 5 min at 4 °C; the supernatant was incubated with anti-cMyc antibodies (Sigma, F1804) to collect the protein-DNA complexes at 4 °C overnight. The precipitated DNA was reversely cross-linked and dissolved in distilled H_2_O. The qPCR analysis was performed as described above with the primers listed in Supplementary Table 3.

### EMSA

The full-length *GRF4* was amplified and fused in-frame into the pGEX-6P-1 vector (Invitrogen) and transformed into *Escherichia coli* Rosetta (Novagen). GST-GRF4 recombinant proteins were purified using Glutathione Sepharose 4B beads (GE Healthcare). In brief, DNA probes (117 bp, 24 bp, and 30 bp) were synthesized (GENEray) (Supplementary Table 3) and labeled using a biotin label kit (Beyotime). GST-GRF4 recombinant proteins were incubated with the purified DNA probes at 4 °C in the EMSA binding buffer (Beyotime). DNA gel shift assays were performed using the LightShift Chemiluminescent EMSA kit (Thermo Fisher Scientific). For the competition, unlabeled oligonucleotides (50-, 100- and 500-fold of labeled probes) were added to the EMSA reactions. This experiment was replicated for three times.

### Phylogenetic and genetic diversity analyses

The resequenced data of 176 rice accessions, including 134 cultivated rice accessions and 42 common wild rice accessions that were collected in Guangxi province, the major distribution place of *Or*-III subgroup^37,46^, were subjected to genetic diversity analysis. Nucleotide diversity (π) in wild rice, *indica*, and *japonica* subspecies, and fixation index (*F*_ST_) values among the pairwise subpopulations (*japonica* to *indica*) were calculated with a 20-k-2-k sliding window using VCFtools^59^. Tajima’s D value in the 2-Mb region spanning *MYB61* was calculated with a 20-kb sliding window using VCFtools. Blast search of helitron in the high-quality genomes of five varieties was performed using the assembly database of the National Center for Biotechnology Information (https://www.ncbi.nlm.nih.gov/assembly) with the accession number GCA_001433935.1 (Nipponbare or NP), GCA_002151415.1 (Shuhui498 or R498), GCA_009797565.1 (Kitaake), GCA_001889745.1 (IR8) and GCA_003865215.1 (93-11 or 9311). The detected sequence variations were summarized in Supplementary Table 2.

Phylogenetic tree of the *MYB61* genomic sequences including the 3-kb promoter, the coding region and 3-kb terminator from the 176 rice accessions and 56 additional *rufipogon* accessions classified into *Or*-I, *Or*-II, and *Or*-III^46^ was constructed using maximum likelihood method with MEGA X^60^ on the basis of a distance matrix with 1000 bootstrap replicates. The SNPs within these genome fragments were filtered by minor allele frequency >5% and missing data rate < 30% to generate high-quality SNP information. Visualization of the generated phylogenetic tree was conducted using an online tool iTOL (https://itol.embl.de).

For haplotype network analysis, DnaSP5^61^ and Arlequin ver3.5^62^ were used to align and sort haplotypes based on the variations of *MYB61* in the 232 rice accessions. PopART software^63^ was used to construct the Templeton, Crandall & Sing (TCS) network of the identified haplotypes to estimate the progenitor haplotype score distribution and the modern haplotype score distribution. The absence of helitron transposon in the promoter regions was identified using Delly^64^.

### Reporting summary

Further information on research design is available in the Nature Research Reporting Summary linked to this article.

## Supplementary information

Supplementary Information

Reporting Summary

## Data Availability

Data supporting the findings of this work are available within the paper and its Supplementary Information files. A reporting summary for this article is available as a Supplementary Information file. The datasets and plant materials generated and analyzed during the current study are available from the corresponding authors upon request. The sequencing data for rice accessions that support the findings in this study can be obtained from NCBI database (PRJNA407820, GCA_001433935.1, GCA_002151415.1, GCA_009797565.1, GCA_001889745.1 and GCA_003865215.1) and EBI database (ERP001143). Source data are provided with this paper.

## Source data

Source Data
