# Supplementary material for: MYB61 is regulated by GRF4 and promotes nitrogen utilization and biomass production in rice
Source: Nat Commun. 2020 Oct 15;11:5219. doi: 10.1038/s41467-020-19019-x (PMC7566476; doi:10.1038/s41467-020-19019-x)
Supplement: Supplementary file 1 — Supplementary Information [file 41467_2020_19019_MOESM1_ESM.pdf]

MYB61 is regulated by GRF4 and promotes nitrogen utilization and biomass production in rice

Gao *et al.*

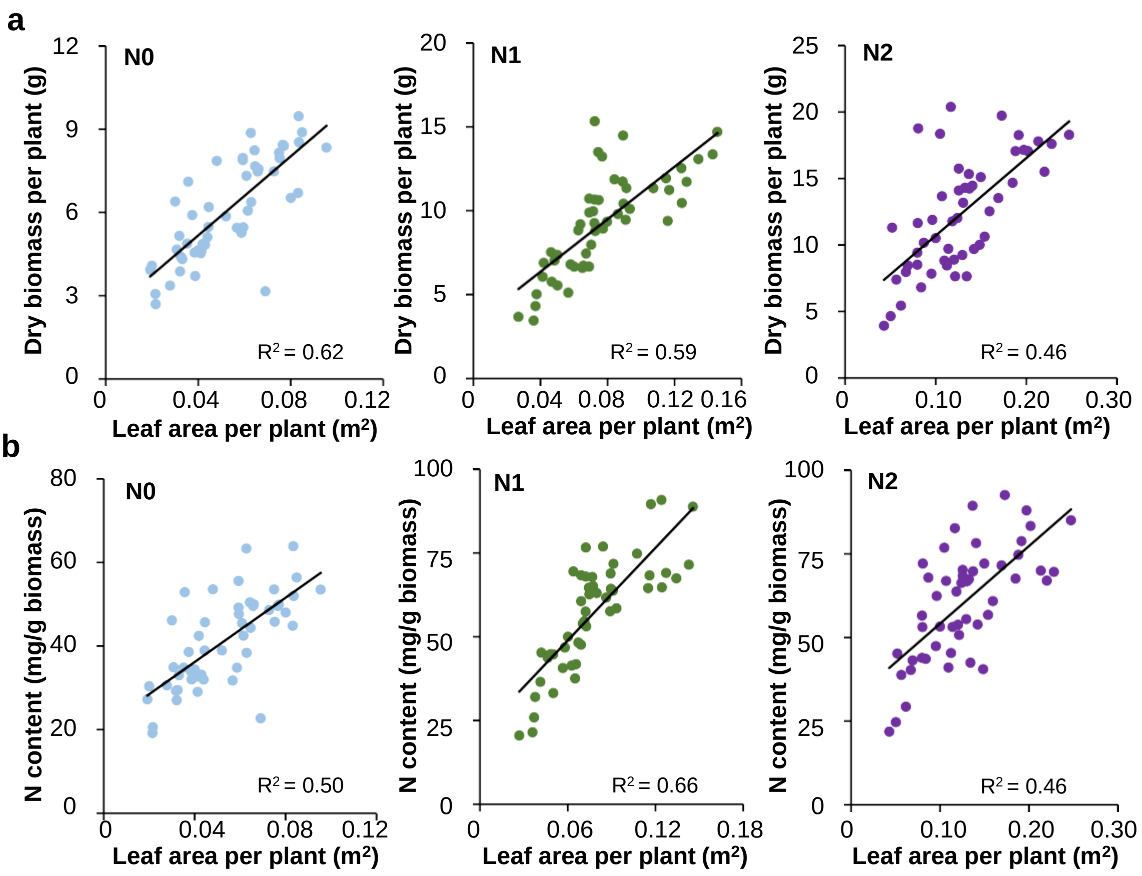

Supplementary Figure 1. Correlation analysis of leaf area with biomass weight (**a**) and the N content (**b**) under different nitrogen supply based on the data shown in Fig. 1**a**−**c**. N0, N1 and N2 indicate 0, 96 and 192 (kg/ha) N fertilizer, respectively. The mean value was obtained from 9 individual plants of each accession. Source data are provided as a Source Data file.

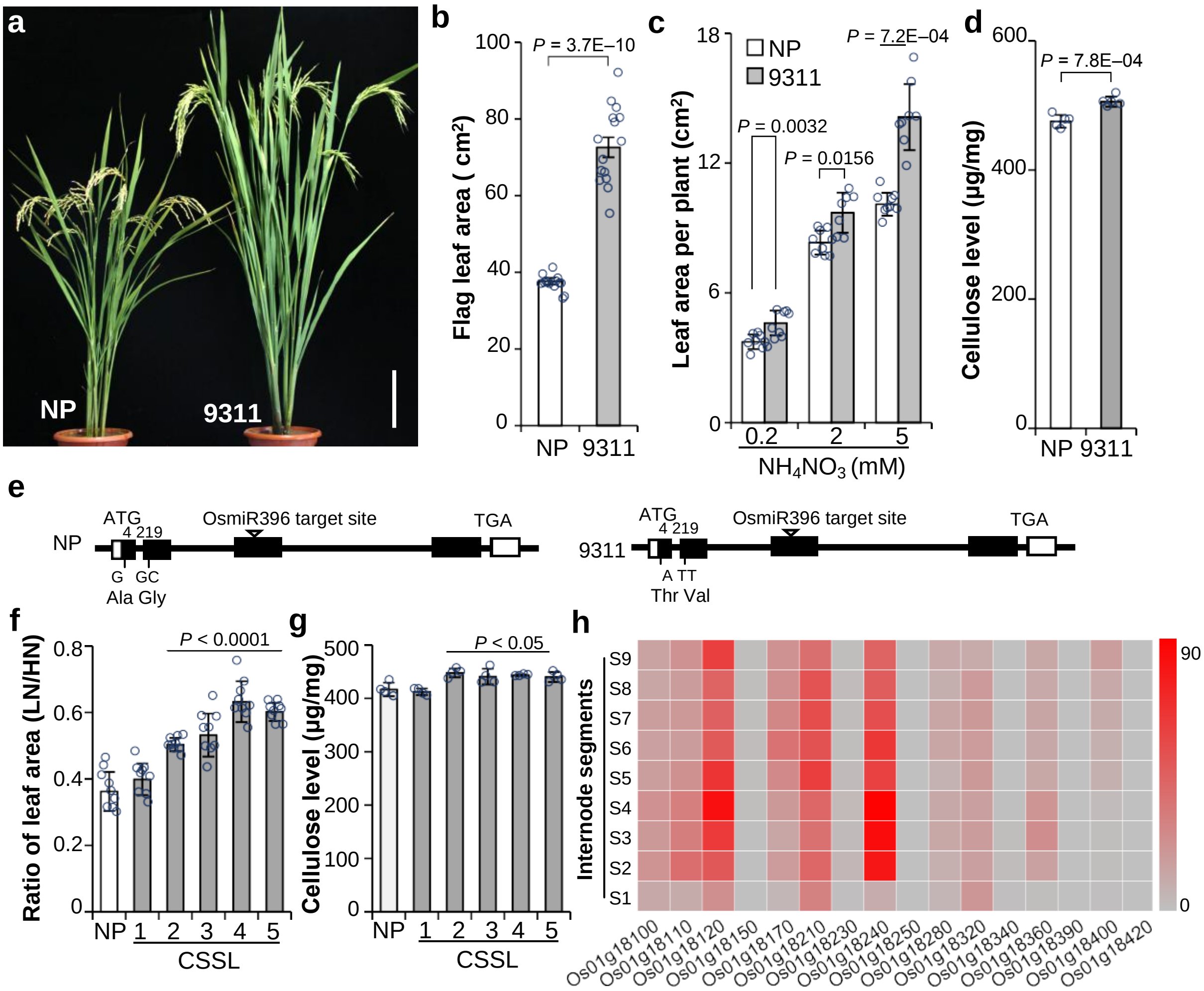

Supplementary Figure 2. Mapping of *MYB61*. **a** Appearance of NP and 9311 mature plants. Bar = 20 cm. **b** Measurement of flag leaf area in mature NP and 9311 growing under 96 kg/ha N condition. Error bars indicate the mean ± SEM of 15 plants. **c** The flag leaf area of NP and 9311 seedlings growing in different NH_4NO_3 supply. Error bars indicate the mean ± SD of at least 8 plants. **d** Measurement of the cellulose content, µg in per mg of cell wall residues prepared from the internodes of mature NP and 9311 plants growing under 96 kg/ha N condition. Error bars indicate the mean ± SD of biological replicates. **e** Schema of the *qNLA2* candidate, showing the *GRF4* gene structure and the variations between NP and 9311. Boxes and lines in the diagram indicate exons and introns, respectively. **f** The ratio of leaf area of NP and the CSSL recombinants (1 to 5) that grew in the media with LN (0.2 mM NH_4NO_3) to that with HN (5 mM NH_4NO_3). Error bars indicate the mean ± SD of 9 plants. **g** The cellulose content, µg in per mg of cell wall residues prepared from the internodes of mature NP and the CSSL plants (1 to 5). Error bars indicate the mean ± SD of 4 biological replicates. **h** The transcript levels of the genes within the fine-mapping region based on the RNA-seq data of the developing 2nd internodes of NP. S1 to S9 indicate the evenly cut internode segments from bottom up. Statistical significance was calculated with two-tailed Welch's unpaired *t*-test and *P* values are indicated in **b-d** and **f-g**. Source data are provided as a Source Data file.

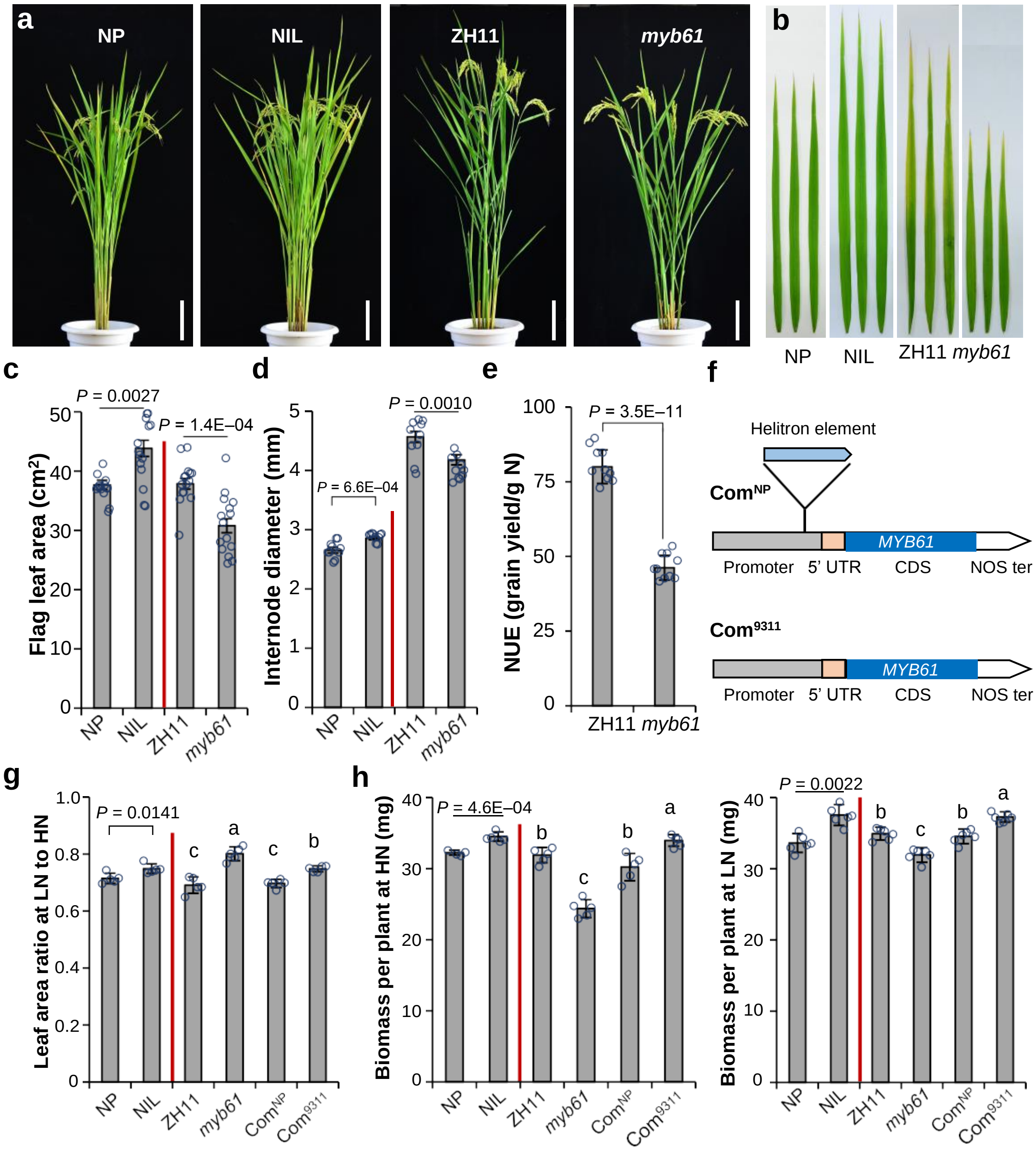

Supplementary Figure 3. Genetic validation of *MYB61*. **a** Mature plants of the NIL and *myb61* mutant and their corresponding wild-type plants. Bar = 20 cm. **b** The flag leaves of the indicated plants. **c** Measurement of the flag leaf area of the indicated plants. Error bars indicate the mean ± SEM of 15 plants. **d** Measurement of 2nd internode diameter of the indicated plants. Error bars indicate the mean ± SEM of 10 plants. **e** Nitrogen use efficiency of ZH11 and *myb61*. Error bars indicate the mean ± SD of 10 biological replicates. **f** The constructs for complementary assay. **g** The ratio of leaf area of the indicated plants growing in the media with LN (0.2 mM NH_4NO_3) to HN (5 mM NH_4NO_3). Error bars indicate the mean ± SD of 5 biological replicates. **h** The biomass amount (dry weight) of the indicated plants growing in HN (5 mM NH_4NO_3, left panel) and LN (0.2 mM NH_4NO_3, right panel). Error bars indicate the mean ± SD of at least 4 biological replicates. a-c in **g** and **h** indicate the different means according to Duncan's multiple range test ($P < 0.05$). Statistical significance was calculated with two-tailed Welch's unpaired *t*-test and *P* values are indicated in **c**-**e**, **g** and **h**. The red vertical lines in this figure are used to separate the plants with different genetic background. Source data are provided as a Source Data file.

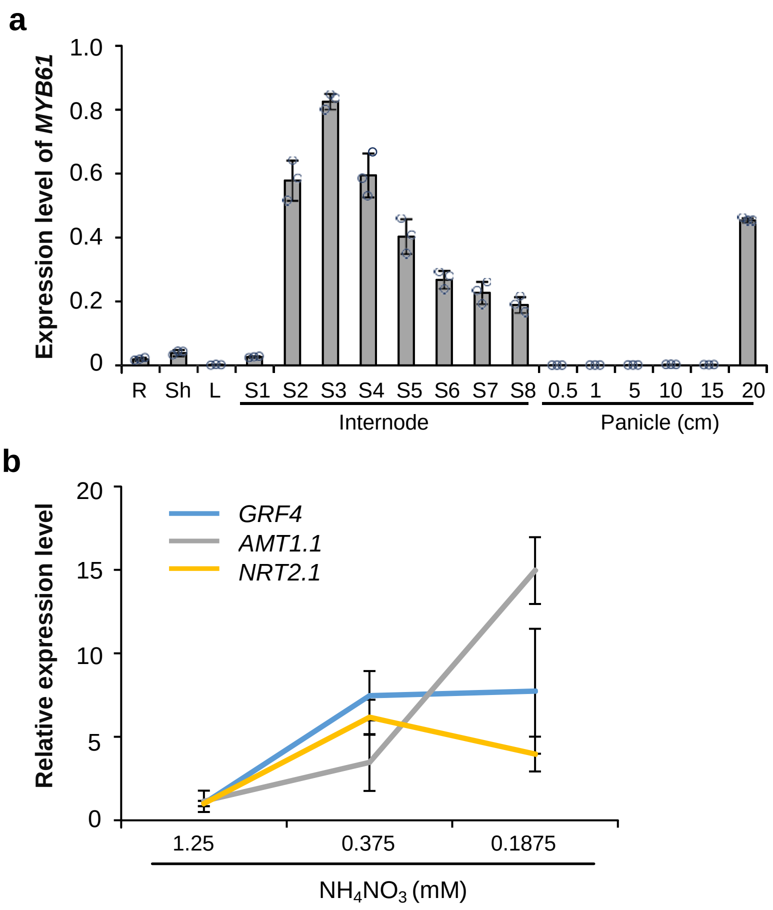

Supplementary Figure 4. Gene expression analysis. **a** Expression profile of *MYB61* in different organs, including the developing internodes and the developing panicles of NP, showing the relative expression levels to rice *HNR*. Error bars represent the mean ± SD of 3 biological replicates. R, root; Sh, leaf sheath; L, leaf. S1 to S8 indicate the evenly cut internode segments from bottom up. **b** Relative expression level of *GRF4*, *AMT1.1* and *NRT2.1* in NP seedlings growing in the media with different concentrations of NH_4NO_3 supply, showing the relative levels to rice *Actin*. Transcription was measured by setting the expression level at 1.25 mM NH_4NO_3 as 1. Error bars represent the mean ± SD of 4 biological replicates. Source data are provided as a Source Data file.

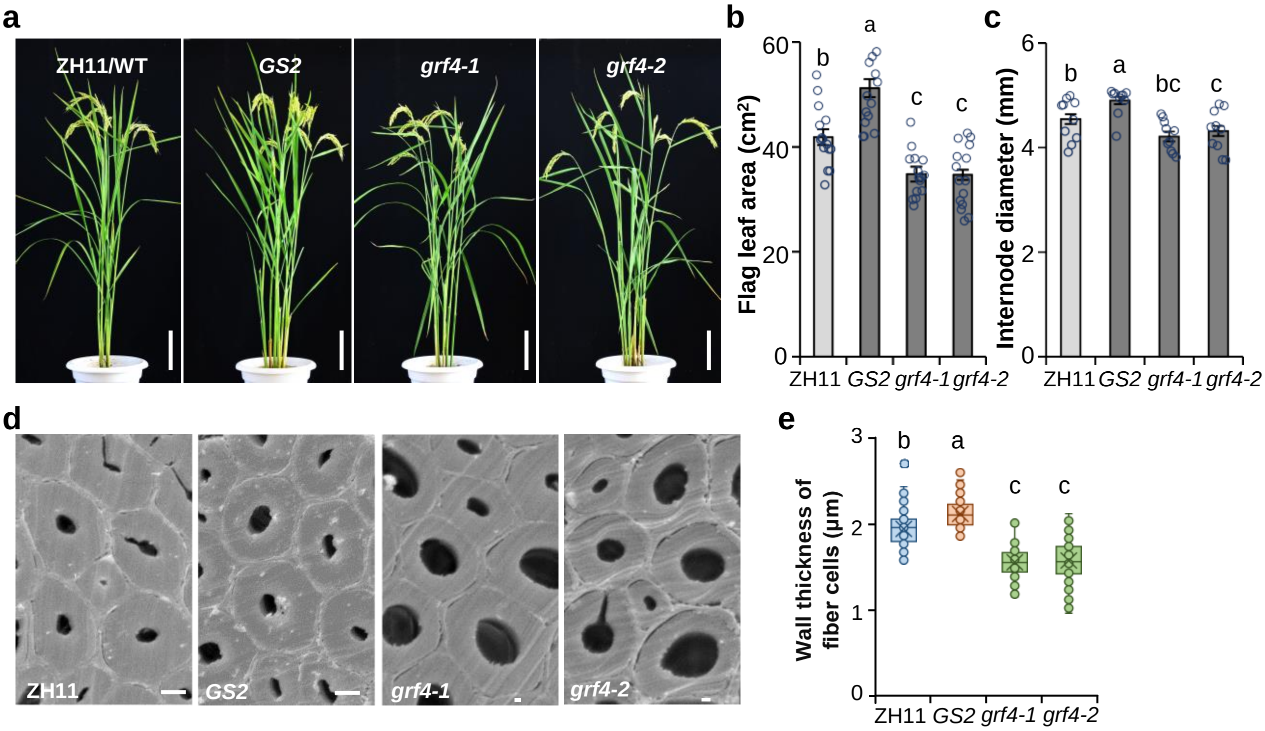

Supplementary Figure 5. GRF4 is essential for cellulosic biomass production. **a** The mature plants of the *grf4* mutants and ZH11/WT. Bar = 15 cm. **b** Measurement of the flag leaf area of the indicated plants. Error bars indicate the mean ± SEM of 15 plants. **c** Measurement of the 2nd internode diameter of the indicated plants. Error bars indicate the mean ± SEM of 10 plants. **d** SEM graphs of sclerenchyma fiber cells from the internodes of the indicated plants. Bars = 2 μm. n = 3 biologically independent samples. **e** Boxplot of the wall thickness of sclerenchyma fiber cells of the indicated plants. Box bounds represent the 25th and 75th percentile, center line represents the median, × indicates the mean, and whiskers represent the 25th percentile − 1.5 * the interquartile range and the 75th percentile + 1.5 * the interquartile range. n = 200 cells from 3 individual internodes of the indicated plants. a-c in **b**, **c** and **e** indicate the different means according to Duncan's multiple range test ($P < 0.05$). Source data are provided as a Source Data file.

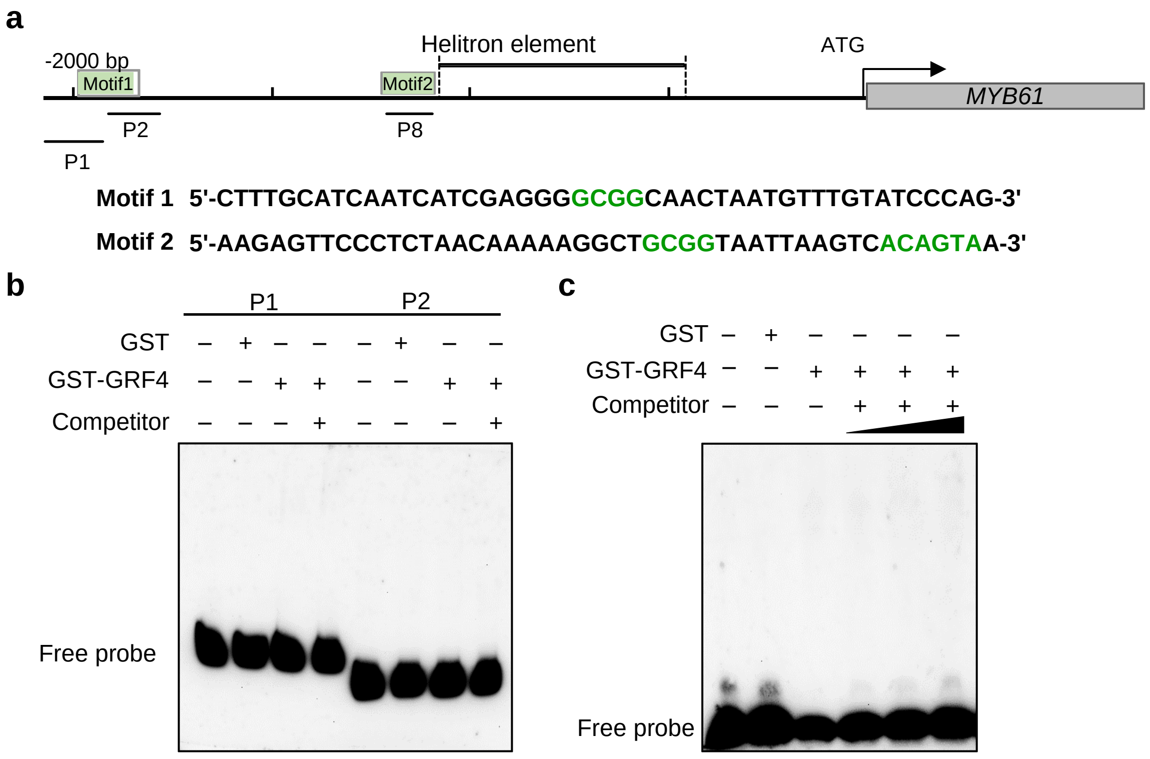

Supplementary Figure 6. Examination of the GRF4 binding motifs in the *MYB61* promoter. **a** Diagram of the 2-kb *MYB61* promoter region. P1, P2 and P8 are the GRF4 association sites shown in **Fig. 4e**. Green boxes indicate the fragment harboring the binding motifs. The DNA sequences containing a core binding motif of GRF4 (the green letters) were subjected to EMSA assay. **b**, **c** EMSA assays, showing no binding between the GRF4 recombinant proteins and the fragments of P1 and P2 (**b**) and the fragment harboring motif 1 (**c**). n = 3 independent experiments. Source data are provided as a Source Data file.

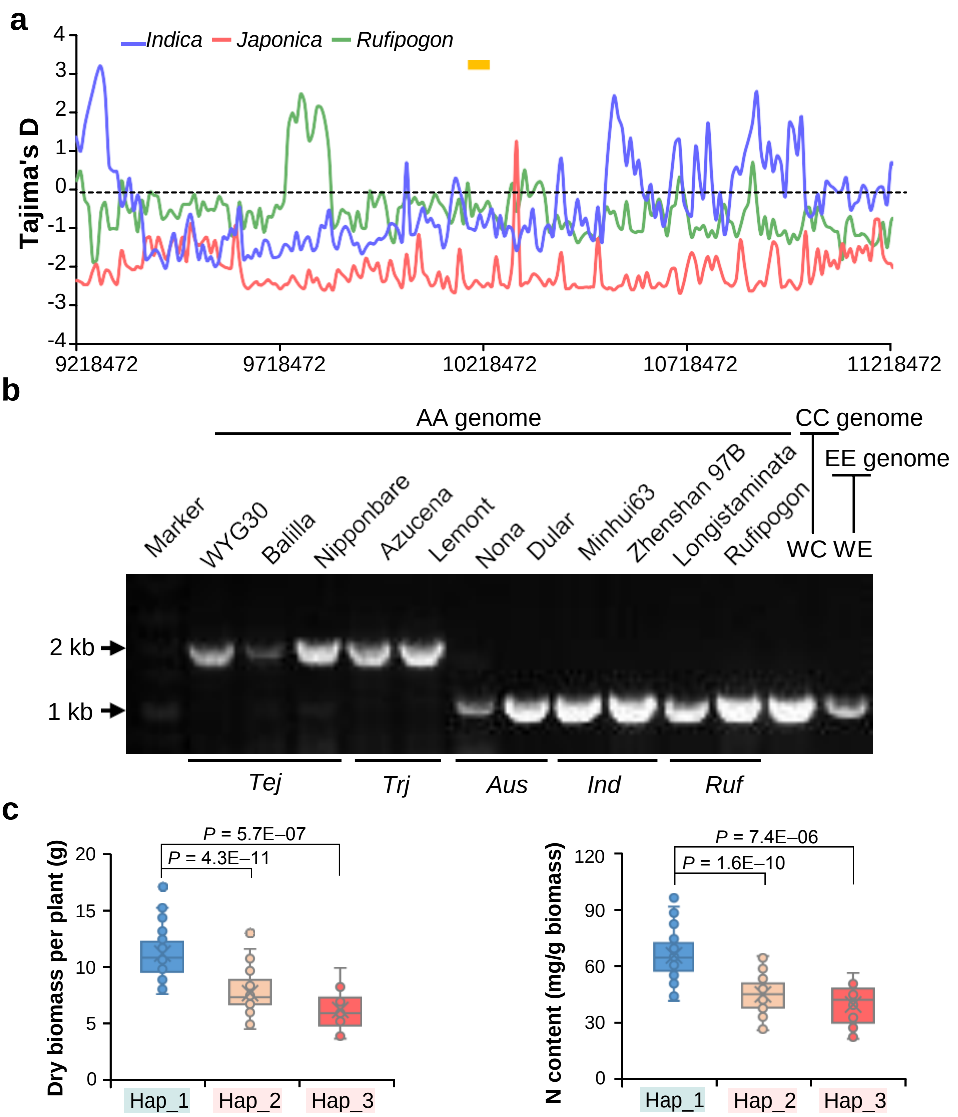

Supplementary Figure 7. Sequence variation analysis of *MYB61*. **a** Tajima's D values between *indica* and *japonica* in the 2000 kb genomic region containing the *MYB61* locus (the yellow line). **b** Analysis of the insertion of the helitron transposon in the rice accessions. *Tej*, *Temperate japonica*; *Trj*, *Tropical japonica*; *Aus*, *Aus* subspecies; *Ind*, *Indica*; *Ruf*, *O. rufipogon*. WC, Wild rice species with CC genome; WE, Wild rice species with EE genome. n = 2 independent experiments. **c** Boxplots of the dry biomass and N content of the rice accessions with haplotype_1 to _3. The mean value was obtained from 9 individual plants of each accession. Box bounds represent the 25th and 75th percentile, center line represents the median, × indicates the mean, and whiskers represent the 25th percentile − 1.5 * the interquartile range and the 75th percentile + 1.5 * the interquartile range. Statistical significance was calculated with two-tailed Welch's unpaired *t*-test and *P* values are indicated. The pink and light blue background indicating haplotypes containing the helitron insertion or not. Source data are provided as a Source Data file.

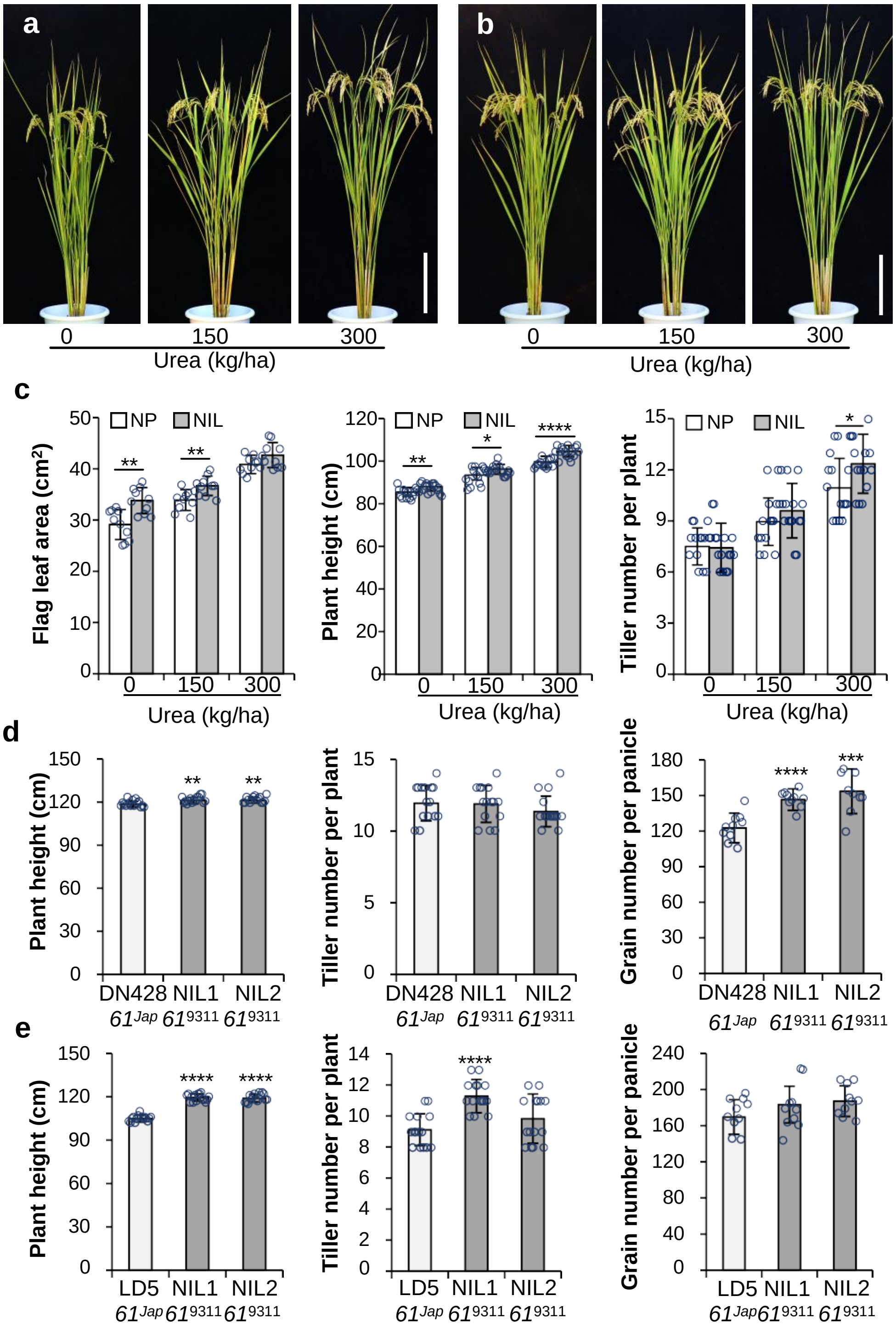

Supplementary Figure 8. *MYB61*^{9311} boosts grain yield in *japonica* varieties. **a**, **b** Appearance of the mature plants of NP (**a**) and the NIL (**b**) growing in the fields with different urea supply. Bar = 25 cm. **c** Measurement of agronomic traits of NP and the NIL under the indicated urea supply. Error bars indicate the mean ± SD of 10 or 15 plants. **d**, **e** Measurement of agronomic traits of the NIL lines in modern *japonica* varieties Dongnong428 (DN428) and Longdao5 (LD5) growing under 150 kg/ha urea supply. Error bars indicate the mean ± SD of 10 or 15 plants. Statistical significance was calculated with two-tailed Welch's unpaired *t*-test and *P* values are indicated (**P* < 0.05, ***P* < 0.01, ****P* < 0.001, *****P* < 0.0001) in **c**−**e**. Source data are provided as a Source Data file.

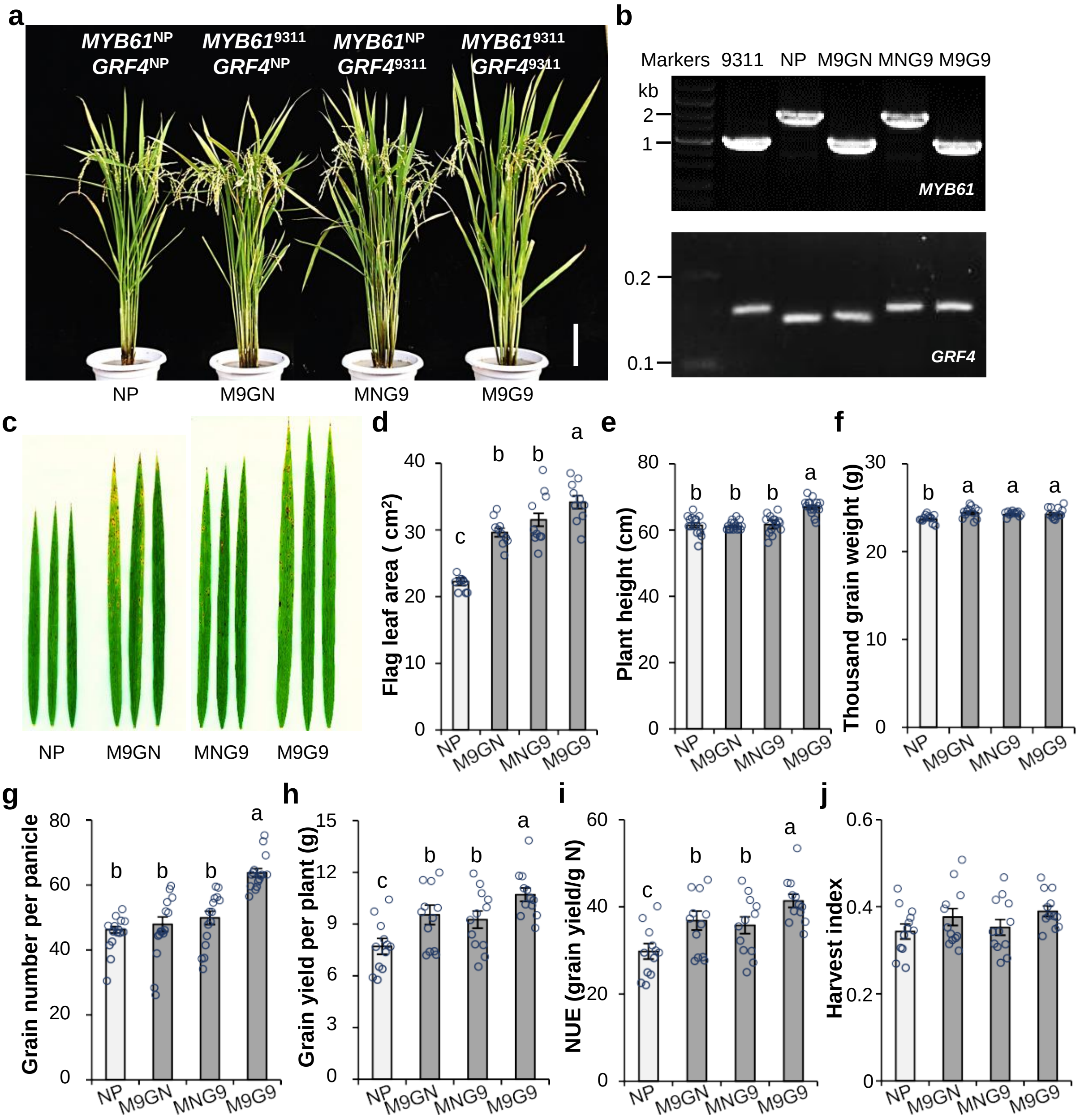

Supplementary Figure 9. Pyramiding *MYB61*^{9311} and *GRF4*^{9311} enhances grain yield. **a** Appearance of the mature plants of NP and the NILs growing in the fields under 150 kg/ha urea condition. Bar = 10 cm. NP, M9GN, MNG9 and M9G9 represent the varied genotypes of *MYB61* and *GRF4* shown with white color letters. **b** Confirming the genotypes of *MYB61* and *GRF4* in the indicated plants with molecular markers. n = 2 independent experiments. **c** Flag leaves of the NIL plants shown in **a**. **d-h** Measurement of agronomic traits of the indicated NIL lines growing under 150 kg/ha urea condition. Error bars indicate the mean ± SEM of 10 plants in **d**, 15 plants in **e** and **g**, 12 plants in **f** and **h**. **i** Measurement of NUE of the indicated NIL lines growing under 150 kg/ha urea condition. Error bars indicate the mean ± SEM of 12 biological replicates. **j** Measurement of harvest index of the indicated NIL lines growing under 150 kg/ha urea condition. Error bars indicate the mean ± SEM of 12 biological replicates. a-c indicate the different means according to Duncan's multiple range test ($P < 0.05$) in **d**-**i**. Source data are provided as a Source Data file.

Supplementary Table 1. The primer sequences used for gene mapping.

Primer name	Primers sequence (5'-3')	
N5-S1	Forward	ACACGGTTTAAAGGTCAAA
	Reverse	ATTCTCTGGCGACGATTT
N5-S2	Forward	GGAAGAGTATGACCAAGA
	Reverse	CTGATTATTAACAAGCACAAC
N5-S3	Forward	GAGATCAGTCAAGACAAC
	Reverse	TGCTTCTCTTCAAATACG
N5-S4	Forward	TACCTACCTCAAGTCAAG
	Reverse	ATCATATCATCAAGTCAACAA
N5-S5	Forward	CGTGGAAGACTTAATGGA
	Reverse	CTAAGCCAAGGTAACTCA
N5-M1	Forward	ACGCATGTCACTAGATTT
	Reverse	TTAGGGTTGGGTTTTATTTG
N5-M2	Forward	ACACGGTTTAAAGGTCAAA
	Reverse	ATTCTCTGGCGACGATTT
N5-M3	Forward	GCCCGTCCTGACTTCTC
	Reverse	TACCATTATCAATGACTTTCT
N5-M4	Forward	CCCACTCACGATAACCTCT
	Reverse	CACCACCCAACACCTAACA
N5-Cel1	Forward	AATCTGAATGGGCTCTAA
	Reverse	GTCAAACAAGAACTGGTAA
N5-M5	Forward	GTGAGCAAGGTAAGGATG
	Reverse	GCTTATGACGAGTCACTC
N5-M6	Forward	GAGCTGATTAATCGGAAG
	Reverse	CAATCATTCATCCATACTACA
N5-M7	Forward	ATTCCTGGTTCTACATTACTTA
	Reverse	CGCCTCACTAGAATATCGGA

Supplementary Table 2. Sequence comparison of the helitron transposon inserted in the *MYB61* promoter of *japonica* in the genome of NP, Kitaake, IR8, R498 and 9311.

Position (i/975)	NP-Hel0065	NP-Chr1_1	NP-Chr1_2	NP-Chr6	Kitaake-Chr1_1	Kitaake-Chr1_2	Kitaake-Chr1_3	Kitaake-Chr6	IR8-Chr8	IR8-Chr12	R498-Chr1_1	R498-Chr1_2	R498-Chr12	9311-Chr8
1	**TC**	AT			AT									
30	**G**	T			T									
59	**A**	G	G	G	G	G		G	G	G	G	G	G	G
91	**G**		A			A								
125	**G**									A			A	
194	**A**													C
248	**G**										A	A		
288	**C**	T			T									
406	**T**			C				C		C			C	
470	**G**											C		
554	**A**	^22 bp	^22bp	^22 bp	^22 bp	^22 bp		^22 bp	^22 bp	^22 bp	^22 bp	^22 bp	^22 bp	^22bp
562	**A**												Del	
613	**T**			C				C		C			C	
629	**A**													G
668	**A**								T					T
716	**A**	G	G	G	G	G		G	G	G	G	G	G	G
730	**C**										A	A		
737	**G**			T				T		T			T	
798	**G**		A			A								
872	**T**			C				C		C			C	
960	**C**											T		

NP-Hel0065 indicates the identified insertional transposon hel_osa_val_0065, which was used as reference sequence (bold blue letters) for alignment. Variations in helitron were listed. The last part of Kitaake_Chr1_3 helitron sequence (555-975) was reconfirmed by PCR sequencing. Del, Deletion. ^ indicates insertion. Source data are provided as a Source Data file.

Supplementary Table 3. The primers used in this study.

Constructs	Primer sequence (5'-3')	
p2GW7-GRF4	Forward	GGGGACAAGTTTGTACAAAAAAGCAGGCTACATGCCTCCCTGTCTCCGGC
	Reverse	GGGGACCACTTTGTACAAGAAAGCTGGGTATCAGTCACCATTAGTTGATCGAG
pUC19-*MYB61p*	Forward	CAGCTATGACCATGATTACGCCAAGCTTAGAAAAACAACATAGCTGGCTGCT
	Reverse	TAGAGGAAGGGTCTTGCGATCCTCTAGATGAATCAATGCAGAAACTGAAG
MYB61p	Forward	AGAAAAACAACATAGCTGGCTGCT
	Reverse	TGAATCAATGCAGAAACTGAAG
MYB61-sgRNA	Forward	gccaGGTGGTCTCAAATTGCAACA
	Reverse	aaacTGTTGCAATTTGAGACCAC
GRF4-sgRNA-T1	Forward	ggcaGGACGCGCCGATGACCGCG
	Reverse	aaacCGCGGTCATCGGCGCGTCC
GRF4-sgRNA-T2	Forward	gccgGCAGGCGCTCATATACAAGT
	Reverse	aaacACTTGTATATGAGCGCCTGC
GRF4-genome	Forward	TTATTGCGCGGCTTGTCT
	Reverse	GTTTGTCGTCTAGCTCGG
ChIP-*MYB61p*-P1	Forward	ATTGGGATTTGCTTCTGAGCGAG
	Reverse	AATCTGTGCTCCAATCCAAG
ChIP-*MYB61p*-P2	Forward	GATCTTCTTGGATTGGAGC
	Reverse	TGTATCCCAGGACAGAAAGAA
ChIP-*MYB61p*-P3	Forward	TTCTTTCTGTCCTGGGATAC
	Reverse	CCAGGCTAAAGTACATACCA
ChIP-*MYB61p*-P4	Forward	TGGTATGTACTTTAGCCTGGC
	Reverse	AACCCAGTTTGCTTGCATCTATC
ChIP-*MYB61p*-P5	Forward	GATAGATGCAAGCAAACTGGG
	Reverse	TCAGCAACCTCTGCGTCA
ChIP-*MYB61p*-P6	Forward	TGACGCAGAGGTTGCTGAGGG
	Reverse	GATTATTGAATGCGACCTG
ChIP-*MYB61p*-P7	Forward	CAGGTCGCATTCAATAATCAC
	Reverse	CTCACCCTTCCTCTACAT
ChIP-*MYB61p*-P8	Forward	CCTTTACTTTTAACTGGCTTGTGC
	Reverse	AATTACCGCAGCCTTTTTGTT
ChIP-*MYB61p*-P9	Forward	TACAACCCCACCGACGTT
	Reverse	AAAGCTGGTGGTTGTGGG
ChIP-*MYB61p*-P10	Forward	ACCACCAGCTTTAAAAGAAGGGGCA
	Reverse	GGGATTGCTGCGAGTGTA
ChIP-*MYB61p*-PC	Forward	GTCACACATCAGATG ATGTTAT
	Reverse	CTTACTTTGTTCCTTGATCTGTTCA
ChIP-*MYB61p*-NC	Forward	CTAGAGGAAGAACTCAAGT
	Reverse	GATACTACTGGAAGATGTTG
EMSA-P2	Forward	ATTGGGATTTGCTTCTGAGCGAG
	Reverse	AATCTGTGCTCCAATCCAAG
EMSA-motif1	Forward	GATCTTCTTGGATTGGAGC
	Reverse	CATCGAGGGGCGGCAACTAATGTT
EMSA-motif2	Forward	CAAAAAGGCTGCGGTAATTAAGTCACAGTA
	Reverse	TACTGTGACTTAATTACCGCAGCCTTTTTG

Supplementary Table 4. The primer sequences used for qRT-PCR analysis.

Primer name	Primers sequence (5'-3')	
qRT-*Os01g18100*	Forward	CTTTCTACCACTTGAGCGAAAC
	Reverse	AAACTCAACACTACCCATGGAT
qRT-*Os01g18110*	Forward	AACTTATGCGATGGTGTTTTCC
	Reverse	GATGACATTCTTTGTTCCCTCG
qRT-*Os01g18120*	Forward	TACTTCGTCTTATGGTGCTGTC
	Reverse	GCAATACCAGTTCTGTGTTTGT
qRT-*Os01g18170*	Forward	TTAGTGGATACTGTCTGTAGCG
	Reverse	CACACAAAACGGAACAAGTCTT
qRT-*Os01g18210*	Forward	CAGAACAACAATGGTACTGGTG
	Reverse	GATATGGGAGAAAGGTTCGAGT
qRT-*Os01g18280*	Forward	CGTCAACGTGCATTGTTACTAT
	Reverse	GATCAAGTACATCAAGCTGCTG
qRT-*Os01g18320*	Forward	GACAGTTGCATCTTTCAGGAAG
	Reverse	TGCTTGTCAACTTCCATGAAAG
qRT-*Os01g18360*	Forward	TTCTCAACTCGATCCATCTCTC
	Reverse	CACTCCTCCATAGCTACGAATG
qRT-*MYB61*	Forward	CTAGAGGAAGAACTCAAGT
	Reverse	GATACTACTGGAAGATGTTG
qRT-*HNR*	Forward	GGCAGGTTCTGCAGTGGTAT
	Reverse	TAAGGTCGGTATCGCCAATC
qRT-*TP1*	Forward	TAACTGGTGCGAACTGCAAG
	Reverse	CGGAGTTGATGATGTCGATG
qRT-*GRF4*	Forward	AGCAGCAGGCGCTCATATAC
	Reverse	TTCTTGCCGAAGTACGGACC
qRT-*AMT1.1*	Forward	CTGGGGTTGGTGGGTTCA
	Reverse	CACTTGGTTGTTGCTGTTGGAG
qRT-*NRT2.1*	Forward	CTTCACGTCGTCGAGGTACT
	Reverse	CACTCGGAGCCGTAGTAGTG
qRT-*Actin*	Forward	ACCATTGGTGCTGAGCGTTT
	Reverse	CGCAGCTTCCATTCCTATGAA
