# Supplementary material for: MYB61 is regulated by GRF4 and promotes nitrogen utilization and biomass production in rice
Source: Nat Commun. 2020 Oct 15;11:5219. doi: 10.1038/s41467-020-19019-x (PMC7566476; doi:10.1038/s41467-020-19019-x)
Supplement: Supplementary file 2 — Reporting Summary [file 41467_2020_19019_MOESM2_ESM.pdf]

nature research

Corresponding author(s): Yihua Zhou

Last updated by author(s): Sep 7, 2020

Reporting Summary

Nature Research wishes to improve the reproducibility of the work that we publish. This form provides structure for consistency and transparency in reporting. For further information on Nature Research policies, see Authors & Referees and the Editorial Policy Checklist .

Statistics

For all statistical analyses, confirm that the following items are present in the figure legend, table legend, main text, or Methods section.

n/a	Confirmed	
☐	☒	The exact sample size (*n*) for each experimental group/condition, given as a discrete number and unit of measurement
☐	☒	A statement on whether measurements were taken from distinct samples or whether the same sample was measured repeatedly
☐	☒	The statistical test(s) used AND whether they are one- or two-sided<br>*Only common tests should be described solely by name; describe more complex techniques in the Methods section.*
☒	☐	A description of all covariates tested
☐	☒	A description of any assumptions or corrections, such as tests of normality and adjustment for multiple comparisons
☐	☒	A full description of the statistical parameters including central tendency (e.g. means) or other basic estimates (e.g. regression coefficient) AND variation (e.g. standard deviation) or associated estimates of uncertainty (e.g. confidence intervals)
☐	☒	For null hypothesis testing, the test statistic (e.g. *F*, *t*, *r*) with confidence intervals, effect sizes, degrees of freedom and *P* value noted<br>*Give P values as exact values whenever suitable.*
☒	☐	For Bayesian analysis, information on the choice of priors and Markov chain Monte Carlo settings
☒	☐	For hierarchical and complex designs, identification of the appropriate level for tests and full reporting of outcomes
☐	☒	Estimates of effect sizes (e.g. Cohen's *d*, Pearson's *r*), indicating how they were calculated

Our web collection on statistics for biologists contains articles on many of the points above.

Software and code

Policy information about availability of computer code

Data collection	Resequencing data of 134 core rice accessions (Nat Genet, 2018, 50, 1435-1441) and 56 wild rice accessions (Nature, 2012, 490, 497–501) were used in this study. No software for data collection was used.
Data analysis	ICIMapping 4.0 was used for QTL analysis; AgriPheno High Throughput Plant Genotyping-Phenotyping-Breeding Service Platform, as well as Easy Leaf Area V2 software, was used to quantify leaf area; VCFtools version 0.1.16 was used to calculate FST values and Tajima's D values; MEGA-X version 10.0.5 and iTOL version 5.6.2 were used to construct and view phylogenetic tree; DnaSP5, Arlequin ver 3.5, and PopART ver 1.7 were used in haplotype network analysis.

For manuscripts utilizing custom algorithms or software that are central to the research but not yet described in published literature, software must be made available to editors/reviewers. We strongly encourage code deposition in a community repository (e.g. GitHub). See the Nature Research guidelines for submitting code & software for further information.

Data

Policy information about availability of data

All manuscripts must include a data availability statement. This statement should provide the following information, where applicable:

- Accession codes, unique identifiers, or web links for publicly available datasets
- A list of figures that have associated raw data
- A description of any restrictions on data availability

Data supporting the findings of this work are available within the paper and its Supplementary Information files. A reporting summary for this Article is available as a Supplementary Information file. The datasets and plant materials generated and analyzed during the current study are available from the corresponding authors upon request. The source data underlying Figures 1, 2d, 3a, d, e, g, h, 4b–f, 5, 6a–d, f–h, as well as Supplementary Figures 1, 2b–d, f–h, 3c–e, g, h, 4, 5b, c, e, 6b, c, 7, 8c–e and 9b, d–j and Supplementary Table 2 are provided as a Source Data file. The sequencing data for rice accessions that support the findings in this study can be obtained from NCBI database (PRJNA407820 [https://www.ncbi.nlm.nih.gov/bioproject/PRJNA407820], GCA_001433935.1 [https://www.ncbi.nlm.nih.gov/assembly/GCF_001433935.1], GCA_002151415.1 [https://www.ncbi.nlm.nih.gov/assembly/GCA_002151415.1], GCA_009797565.1 [https://www.ncbi.nlm.nih.gov/assembly/GCA_009797565.1], GCA_001889745.1 [https://www.ncbi.nlm.nih.gov/assembly/GCA_001889745.1/] and GCA_003865215.1 [https://

www.ncbi.nlm.nih.gov/assembly/GCA_003865215.1]) and EBI database (ERP001143, https://www.ebi.ac.uk/ena/browser/view/PRJEB2829)

Field-specific reporting

Please select the one below that is the best fit for your research. If you are not sure, read the appropriate sections before making your selection.

[x] Life sciences [ ] Behavioural & social sciences [ ] Ecological, evolutionary & environmental sciences

For a reference copy of the document with all sections, see nature.com/documents/nr-reporting-summary-flat.pdf

Life sciences study design

All studies must disclose on these points even when the disclosure is negative.

Sample size	For field trial assay and nitrogen treatments, 9 individual plants of each accession were quantified. For evaluation of yield and nitrogen use efficiency, 3 × 25 plants of the tested varieties and 12-15 individual plants were used. No statistical methods were used to predetermine sample sizes.
Data exclusions	No data were excluded from our analyses.
Replication	For the experiments like EMSA, ChIP-PCR, and transactivation activity assays, at least three independent experiments were conducted. For cellulose content measurement, four biological replicates were included for each samples. For gene expression analyses, at least three independent experiments were performed. For measurement of cell wall thickness, 200 cells from the internodes of three biologically independent plants were examined. For genotyping MYB61 in the indicated plants, at least two independent experiments were performed.
Randomization	The experiments were used to compare phenotypes among wild type, mutants, and transgenic lines, therefore, sample allocation is not relevant to this study.
Blinding	The experiments were used to compare phenotypes among wild type, mutants, and transgenic lines, therefore, blinding is not relevant to this study.

Reporting for specific materials, systems and methods

We require information from authors about some types of materials, experimental systems and methods used in many studies. Here, indicate whether each material, system or method listed is relevant to your study. If you are not sure if a list item applies to your research, read the appropriate section before selecting a response.

Materials & experimental systems

n/a	Involved in the study	
[ ]	[x]	Antibodies
[x]	[ ]	Eukaryotic cell lines
[x]	[ ]	Palaeontology
[x]	[ ]	Animals and other organisms
[x]	[ ]	Human research participants
[x]	[ ]	Clinical data

Methods

n/a	Involved in the study	
[x]	[ ]	ChIP-seq
[x]	[ ]	Flow cytometry
[x]	[ ]	MRI-based neuroimaging

Antibodies

Antibodies used	anti-cMyc antibodies (Sigma, F1804) was used for ChIP-PCR analysis.
Validation	Brizzard, B.L., et al., Immunoaffinity purification of FLAG epitope-tagged bacterial alkaline phosphatase using a novel monoclonal antibody and peptide elution. BioTechniques, 16, 730-735 (1994).
